# Genetic structure and polymorphisms of Gelao ethnicity residing in southwest china revealed by X-chromosomal genetic markers

**DOI:** 10.1038/s41598-018-32945-7

**Published:** 2018-10-01

**Authors:** Pengyu Chen, Guanglin He, Xing Zou, Mengge Wang, Haibo Luo, Limei Yu, Xijie Hu, Mijia Xia, Hongyan Gao, Jian Yu, Yiping Hou, Yanyan Han

**Affiliations:** 1 Center of Forensic Expertise, Affiliated Hospital of Zunyi Medical University, Zunyi, Guizhou China; 2 Department of Forensic Medicine, Zunyi Medical University, Zunyi, Guizhou China; 3 Institute of Forensic Medicine, West China School of Basic Medical Sciences & Forensic Medicine, Sichuan University, Chengdu, Sichuan China; 4 Key Laboratory of Cell Engineering in Guizhou Province, Zunyi, Guizhou China; 5 Judicial Authentication Institution, First People’s Hospital of Zunyi City, Zunyi, Guizhou China; 6 Department of Nutrition and Food Hygiene, School of Public Health, Zunyi Medical University, Zunyi, Guizhou China

## Abstract

X-chromosome short tandem repeat markers (X-STRs), due to their special inheritance models, physical location on a single chromosome and the absence of recombination in male meiosis, play an important role in forensic and population genetics. While a series of genetic analyses focusing on the genetic diversity and forensic characteristics of X-STRs are well studied for ethnically/linguistically diverse and demographically large Chinese populations, genetic evidence from Gelao ethnicity is still sparse. Here, we genotyped the first batch of 19 X-STRs in 513 Chinese Gelao individuals (265 females and 248 males), and reported genetic polymorphisms, forensic characteristics based on the single locus and seven linkage groups. DXS10135 with the highest PIC (0.9106) and LG1 (DXS10148-DXS10135-DXS8378) with the largest HD (0.9970) are polymorphic and informative. The CPDs in Gelao males and females are respectively larger than 0.999999999997095 and 0.99999999999999999999918, and the combined MECs are larger than 0.999999975715109. Subsequently, we investigated the population relationships among 14 Chinese populations based on 19 X-STRs and among 23 populations based on 11 overlapped X-STRs. Our results revealed genetic differentiations among Tibeto-Burman, Altaic and other Chinese homogenous populations, and demonstrated that Guizhou Gelao has the genetically closer relationships with Han Chinese and geographically close Guizhou Miao.

## Introduction

Short tandem repeat (STR), one kind of mutation-prone genetic marker and also often referred to as microsatellite and simple sequence repeat (SSR), is widely distributed in the human genome (approximately 1.6 million and spanning nearly 1% of the human genome)^1–3^. STR is the repetitive nucleotide sequence, which comprises a repeating motif of 2–6 base pairs^2^. Previous studies have suggested that slippage events during the DNA replication make the contribution to higher mutation rate of averagely 10^−3^ to 10^−4^ mutations per generation than other types of genetic markers, such as binary markers of single nucleotide polymorphisms and insertion/deletions^4,5^. A large-scale surveys focused on lager number of autosomal STR variations have been performed and demonstrated that STRs are associated with regulating gene expression and complex molecular phenotype traits, as well as prevalence and susceptibility of Mendelian diseases and cancers^4–7^. Y-chromosomal STRs with the features of high mutation and male especial inheritance play an important role in the population genetics, genealogy researches, evolutionary and forensic studies^8,9^. In forensic science, more attentions have been paid to widely in the rates and patterns of de novo STR mutations, genetic polymorphisms and forensic characteristics of the CODIS (Combined DNA Index System) or expanded CODIS markers^10–14^, or specific Y chromosome STRs (Y Filer Plus and PowerPlex Y23)^15–18^ in geographically, linguistically, and ethnically diverse populations^9,19–21^.

X-chromosomal STRs with the advantage features of autosomal and uniparental biomarkers have been recognized to play an important complementary role in forensic deficiency cases and other complex kinship identifications^22^. In the past decade, the commercially available kits of Mentype Argus X-8 kit^23^ (Biotype, Dresden, Germany) and Investigator Argus X-12^24^ (Qiagen, Hilden, Germany) which can respectively co-amplify eight X-STRs and twelve X-STRs belonging to four linkage groups were utilized in human identification purposes and complex kinship identifications. Recently, to get higher discriminatory power, a new commercial AGCU X19 X-STRs amplification kit (AGCU ScienTech Inc., Wuxi, Jiangsu, China) has been developed^25^ and emerged to characterize the genetic polymorphisms and forensic characteristics, as well as reconstruct X-chromosomal genetic marker haplotype reference database in Chinese nationalities^26–31^. Unfortunately, genetic variations, haplotype diversity of X-chromosomal genetic markers in Chinese Gelao, as well as genetic relationships with geographically/ethnolinguistically related populations have not yet been addressed.

China is one ethnolinguistically diverse country consisting of 55 minority ethnic groups and one world largest group of Han nationality, whose speaking languages belong to at least five language families (Tai-Kadai, Sino-Tibetan, Austroasiatic, Austronesian, and Hmong-Mien). Each of Chinese ethnicities is enriched with the special and complex population history, including origin, migration, as well as cultural and genetic admixture^32–35^. Recently, China has been recognized as one of the hub of geneticists, molecular anthropologists, linguists, and archeologists^32–35^. Gelao ethnic group with the total population over 0.55 million, mainly scattered in the provinces of Guizhou, Guangxi, Sichuan, and Yunnan, as well as Ha Giang in northern Vietnam. And over 96% of Chinese Gelaos reside in Guizhou according to 2012 census. Previous cultural and archeological evidence has shown that present Gelao people are descendants of ancient Liao people residing in southwest China (https://en.wikipedia.org/wiki/Gelao_people). Ancient Liao, as a typical slow development population due to the limitations of the availability of resources, disgusting climatic conditions, possible diseases, and the spread of technological and cultural innovations, has experienced the long history of Hunter-Gather and Agriculture periods with hunter-gathering and nomadic lifestyle^15,36,37^.

In the present study, we first genotyped the 19 X-STR loci in 513 Chinese Gelao individuals and then integrated our data with 13 previously published populations^26–31,38–40^ based on genetic variations of 19 X-STRs, and with 22 populations^26–31,38–49^ on the basis of 11 overlapped STR loci between the AGCU X19 amplification system^25^ and Investigator Argus X-12 amplification kit^42^. We sought out to address the following questions: (1) what about the genetic diversity of 19 X-STRs and seven linkage groups in Gelao ethnicity? (2) what are the locations of Chinese Gelao ethnicity in a nationwide genetic variation context? (3) what are the linguistic, geographic and social affiliations based on X-chromosomal genetic markers? (4) what are the features of Chinese population genetic substructures and the genetic distances between Gelao nationality and other reference populations?

## Results

### Hardy-Weinberg equilibrium, linkage disequilibrium and gender differentiation

In the present study, we successfully genotyped 19 X-chromosomal STRs in 513 Chinese Gelao individuals (265 females and 248 males) residing in Guizhou province, southwestern China. Linkage disequilibrium (LD) for all 171 pairs of loci among female individuals was conducted by permutation test using the expectation-maximization (EM) algorithm with the number of permutations of 10,000 and initial conditions for EM of 2, and exact test of pairwise LD in 248 male individuals was performed employing a Markov chain with the chain length of 10,000 and dememorization of 1000^50^. Statistically significant deviations from LD expectation are observed in 8 pairwise comparisons (DXS8378-DXS10134, DXS10134-HPRTB, DXS10079-DXS6809, DXS10103-DXS10101, HPRTB-DXS6809, and DXS6809-DXS10135) in the female individuals (Supplementary Table S1). However, no deviations are observed except DXS10103-DXS10101 (p = 0.0000) after Bonferroni correction (p > 0.05/171 = 0.0003). In male population, DXS10134 with four loci (DXS7423, DXS10148, DXS10159 and DXS10101), DXS10164 with two loci (DXS8378 and DXS10162), DXS10162 with two loci (DXS10159 and DXS10164), DXS101 with DXS7424, DXS10101 with three loci (DXS10134, DXS10103 and DXS10135), DXS6809 with three loci (DXS7424, DXS10103 and DXS10135), DXS10075 with two loci (DXS10103 and DXS10135), and DXS10135 with three loci (DXS10101, DXS6809 and DXS10075) are observed with significant deviations from the LD. Only remaining four pairs (DXS8378-DXS10164, DXS10134-DXS10148, DXS101-DXS7424, and DXS10101-DXS10103) are still deviated from the LD after Bonferroni correction of the multiple test.

We next performed the exact test using the Markov Chain with the forecasted chain length of 1,000,000 and dememorization steps of 100,000 to examine the Hardy-Weinberg equilibrium (HWE) of 19 X-STRs in the 265 female individuals on the basis of the distributions of the observed heterozygosity (Ho) and expected heterozygosity (He)^51^. As shown in Table 1, the values of Ho and He span the ranges between 0.5019 (DXS7423) and 0.9019 (DXS10135), and 0.5433 (DXS7423) and 0.9158 (DXS10135), respectively. No deviations from the HWE are observed with the exception of DXS10134 (p = 0.0360). After applying the Bonferroni correction (p = 0.0026), all tested X-STRs are in conformity with the HWE. The allele frequencies of Gelao females and males are presented in the Supplementary Tables S3, S4 and Figs S1, S2. A total of 229 alleles with corresponding allelic frequencies ranging from 0.0019 to 0.5736 in females, and 201 alleles with corresponding allelic frequencies spanning from 0.0040 to 0.6169 in males are observed. The Fst and corresponding p values were calculated to explore the gender differentiations among female and male samples using the exact test in the locus-by-locus comparison and presented in Supplementary Table S5. Considering that no significant statistical differences between males and females are observed, we pooled the male and female samples to recalculate the allele frequency distributions and forensic statistical parameters. As shown in Supplementary Table S6 and Fig. S3, a total of 242 alleles are identified with corresponding frequencies ranging from 0.013 to 0.5874.

**Table 1:** Forensic parameters of Gelao population based on the genetic variability of 19 X-STRs.

Loci	Number of alleles	Ho	He	p	GD	PIC	PD_m_	PD_f_	MEC Krüger	MEC Kishida	MEC Desmarais	MEC Desmarais duo
DXS8378	9	0.6679	0.6149	0.4440	0.6124	0.5502	0.6116	0.7878	0.3528	0.5502	0.5502	0.4034
DXS7423	5	0.5019	0.5433	0.2801	0.5441	0.4548	0.5434	0.7030	0.2607	0.4551	0.4548	0.3162
DXS10148	22	0.8868	0.9049	0.9841	0.9050	0.8959	0.9038	0.9828	0.8053	0.8959	0.8959	0.8185
DXS10159	10	0.7811	0.7827	0.6685	0.7844	0.7510	0.7834	0.9207	0.5802	0.7511	0.7510	0.6214
DXS10134	21	0.8415	0.8531	0.0360	0.8551	0.8376	0.8540	0.9622	0.7093	0.8375	0.8376	0.7339
DXS7424	9	0.7170	0.7295	0.2950	0.7255	0.6811	0.7246	0.8806	0.4952	0.6811	0.6811	0.5411
DXS10164	10	0.6038	0.6134	0.0831	0.5999	0.5595	0.5992	0.7997	0.3766	0.5595	0.5595	0.4110
DXS10162	13	0.7736	0.7627	0.9134	0.7626	0.7251	0.7617	0.9066	0.5471	0.7252	0.7251	0.5907
DXS7132	8	0.7321	0.7622	0.5548	0.7562	0.7166	0.7552	0.9014	0.5347	0.7166	0.7166	0.5805
DXS10079	10	0.7698	0.8109	0.5465	0.8056	0.7775	0.8045	0.9347	0.6184	0.7778	0.7775	0.6542
DXS6789	12	0.8377	0.8121	0.9058	0.8147	0.7890	0.8136	0.9407	0.6353	0.7892	0.7890	0.6689
DXS101	14	0.8076	0.8025	0.3278	0.8103	0.7845	0.8093	0.9388	0.6305	0.7847	0.7845	0.6636
DXS10103	10	0.7547	0.7830	0.0566	0.7738	0.7376	0.7728	0.9132	0.5614	0.7379	0.7376	0.6051
DXS10101	20	0.9019	0.8935	0.3214	0.8920	0.8810	0.8908	0.9783	0.7803	0.8810	0.8810	0.7962
HPRTB	7	0.6943	0.7171	0.5692	0.7220	0.6742	0.7211	0.8754	0.4813	0.6741	0.6742	0.5324
DXS6809	10	0.7698	0.8294	0.0808	0.8320	0.8095	0.8310	0.9499	0.6641	0.8094	0.8095	0.6952
DXS10075	14	0.6830	0.7141	0.0999	0.7071	0.6607	0.7062	0.8682	0.4707	0.6608	0.6607	0.5180
DXS10074	12	0.7434	0.7838	0.5779	0.7835	0.7507	0.7825	0.9209	0.5807	0.7508	0.7507	0.6210
DXS10135	26	0.8981	0.9158	0.1347	0.9177	0.9106	0.9166	0.9871	0.8319	0.9108	0.9106	0.8418

Ho, observed heterozygosity in females; He, expected heterozygosity in females; p, p values for Hardy-Weinberg test in female samples; GD, gene diversity; PIC, polymorphism information content; PD_f_, power of discrimination in females; PD_m_, power of discrimination in males; MEC Krüger, mean paternity exclusion chance for autosomal STR markers in trios and complex kinship cases; MEC Kishida, mean paternity exclusion chance for X-chromosomal markers in trios involving daughters; MEC Desmarais, mean paternity exclusion chance for X-chromosomal markers in trios involving daughters (Desmarais version); MEC Desmarais Duo, Mean paternity exclusion chance for X-chromosomal markers in father/daughter duos.

### Forensic parameters of 19 X-STRs based on allele frequency distribution

**Figure 1:**
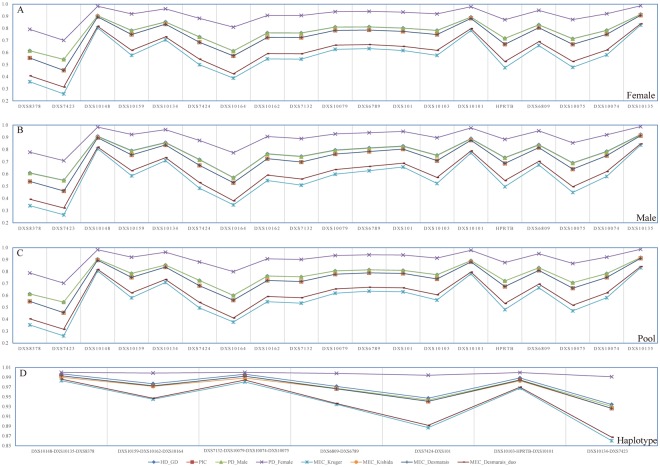
Forensic statistical parameters of Guizhou Gelao population. (**A**) Forensic parameters of 19 X-STRs in Guizhou Gelao female population. (**B**) Forensic parameters are calculated based on the genetic polymorphism of 19 X-chromosomal markers in male population. (**C**) Forensic parameters in Gelao ethnicity are estimated on the basis of pooled allele frequency distributions of males and females. (**D**) Forensic parameters of seven linkage groups are evaluated according to the haplotype frequency distributions.

The forensic parameters, including genetic diversity (GD), polymorphism information content (PIC), power of exclusion (PE), power of discrimination in female (PD_f_) and male (PD_m_), and four mean paternity exclusion change indexes introduced respectively by Krüger *et al*.^52^ (MEC Krüger), Kishida *et al*.^53^ (MEC Kishida), and Desmarais *et al*.^54^ (MEC Desmarais and MEC Desmarais Duo), of Gelao females, males and pooled population are presented in Supplementary Tables 1, S7 and S8, and Fig. 1. In Gelao females, DXS10135 is the most polymorphic and informative locus, in which 23 alleles and the largest aforementioned forensic parameters are identified. However, only 4 alleles are observed at the locus of DXS7423 with the lowest values of forensic statistical indexes (Fig. 1 and Supplementary Table S7). The combined PD_m_ and PD_f_ are 0.99999999999985 and 0.99999999999999999999974. Four combined MEC values are respectively 0.999999975595042, 0.999999999998348, 0.999999999998334, and 0.999999995565582. We subsequently evaluated forensic efficiency in Gelao males. As shown in Fig. 1 and Supplementary Table S8, DXS7423 and DXS8378, with five alleles and the lowest forensic parameter values, are less informative than others. DXS10135 with 22 alleles is the most informative locus, which is consistent with the features observed in the Gelao females. The combined powers of PDM, PDF, MEC Krüger, MEC Kishida, MEC Desmarais and MEC Desmarais Duo are 0.999999999999808, 0.99999999999999999999959, 0.999999970202144, 0.999999999997809, 0.999999999997814, and 0.999999994597184, respectively. Finally, overall forensic features are evaluated (Fig. 1 and Table 1). The largest and smallest informative loci are separately DXS10135 (26 alleles) and DXS7423 (5 alleles). The GD varies from 0.5441 to 0.9177, and PIC spans from 0.4548 to 0.9106. The PDF and PDM span from 0.7030 to 0.9871, and from 0.5434 to 0.9166, respectively. MEC Krüger, MEC Kishida, MEC Desmarais and MEC Desmarais Duo accordingly range from 0.2607 to 0.8319, from 0.4551 to 0.9180, from 0.4548 to 0.9106, from 0.3162 to 0.8418, respectively.

### Forensic parameters of seven linkage groups based on haplotype frequency distribution

19 X-STRs can be grouped into seven linkage groups (LG): LG1 (DXS10148-DXS10135-DXS8378)^55,56^, LG2 (DXS10159-DXS10162-DXS10164)^57^, LG3 (DXS7132-DXS10079-DXS10074-DXS10075)^49^, LG4 (DXS6809-DXS6789)^58^, LG5 (DXS7424-DXS101)^59^, LG6 (DXS10103-HPRTB-DXS10101)^56^ and LG7 (DXS10134-DXS7423)^49^. The haplotype compositions and corresponding haplotype frequencies of seven linkage groups in 248 males are presented in Supplementary Table S9 and Figs S4, S5. A total of 179, 92, 179, 54, 45, 123 and 36 different haplotypes are respectively found in the LG1 to LG7, in which 128, 49, 140, 12, 11, 75 and 11 are unique. The fractions of unique haplotype range from 0.2222 (LG4) to 0.7821 (LG3). The most common haplotypes are 24.1-19-10, 24.1-22-10, 25.1-25-10, 26.1-20-10, and 26.1-21-10 in the LG1 (0.0161), 25-19-10 in the LG2 (0.0726), 13-20-17-17 in the LG3 (0.0242), 33–20 in the LG4 (0.0605), 16–24 in the LG5 (0.1210), 16-13-31 in the LG6 (0.0524), 37–15 in the LG7 (0.1331). The forensic parameters of aforementioned linkage groups are presented in Table 2. The match probabilities span from 0.0070 in the LG1 to 0.0694 in the LG7, and haplotype diversities range from 0.9344 (LG7) to 0.9970 (LG1). The LG1 with the largest forensic parameter is the most informative group and LG7 is the least polymorphic.

**Table 2:** Forensic parameters of seven linkage groups on the basis of the haplotype frequencies in Guizhou Gelao population.

Forensic Parameters	LG1	LG2	LG3	LG4	LG5	LG6	LG7
Number of Haplotypes	179	92	179	54	45	123	36
Fraction of unique haplotype	0.7151	0.5326	0.7821	0.2222	0.2444	0.6098	0.3056
HD	0.9970	0.9769	0.9962	0.9713	0.9473	0.9886	0.9344
MP	0.0070	0.0271	0.0078	0.0326	0.0565	0.0153	0.0694
PIC	0.9929	0.9723	0.9921	0.9664	0.9408	0.9844	0.9264
PD_m_	0.9930	0.9729	0.9922	0.9674	0.9435	0.9847	0.9306
PD_f_	0.9999	0.9986	0.9999	0.9979	0.9941	0.9995	0.9910
MEC Krüger	0.9830	0.9451	0.9805	0.9347	0.8871	0.9677	0.8603
MEC Kishida	0.9900	0.9715	0.9883	0.9667	0.9409	0.9828	0.9264
MEC Desmarais	0.9929	0.9723	0.9921	0.9664	0.9408	0.9844	0.9264
MEC Desmarais duo	0.9860	0.9471	0.9845	0.9362	0.8918	0.9697	0.8675

HD, haplotype diversity; MP, march probability; PIC, polymorphism information content; PD_f_, power of discrimination in females; PD_m_, power of discrimination in males; MEC Krüger, mean paternity exclusion chance for autosomal STR markers in trios and complex kinship cases; MEC Kishida, mean paternity exclusion chance for X-chromosomal markers in trios involving daughters; MEC Desmarais, mean paternity exclusion chance for X-chromosomal markers in trios involving daughters (Desmarais version); MEC Desmarais Duo, Mean paternity exclusion chance for X-chromosomal markers in father/daughter duos. LG1: DXS10148-DXS10135-DXS8378; LG2: DXS10159-DXS10162-DXS10164; LG3: DXS7132-DXS10079-DXS10074-DXS10075; LG4: DXS6809-DXS6789; LG5: DXS7424-DXS101; LG6: DXS10103-HPRTB-DXS10101; LG7: DXS10134-DXS7423.

### Comprehensive population comparisons based on 19 X-STRs among 14 Chinese populations

**Figure 2:**
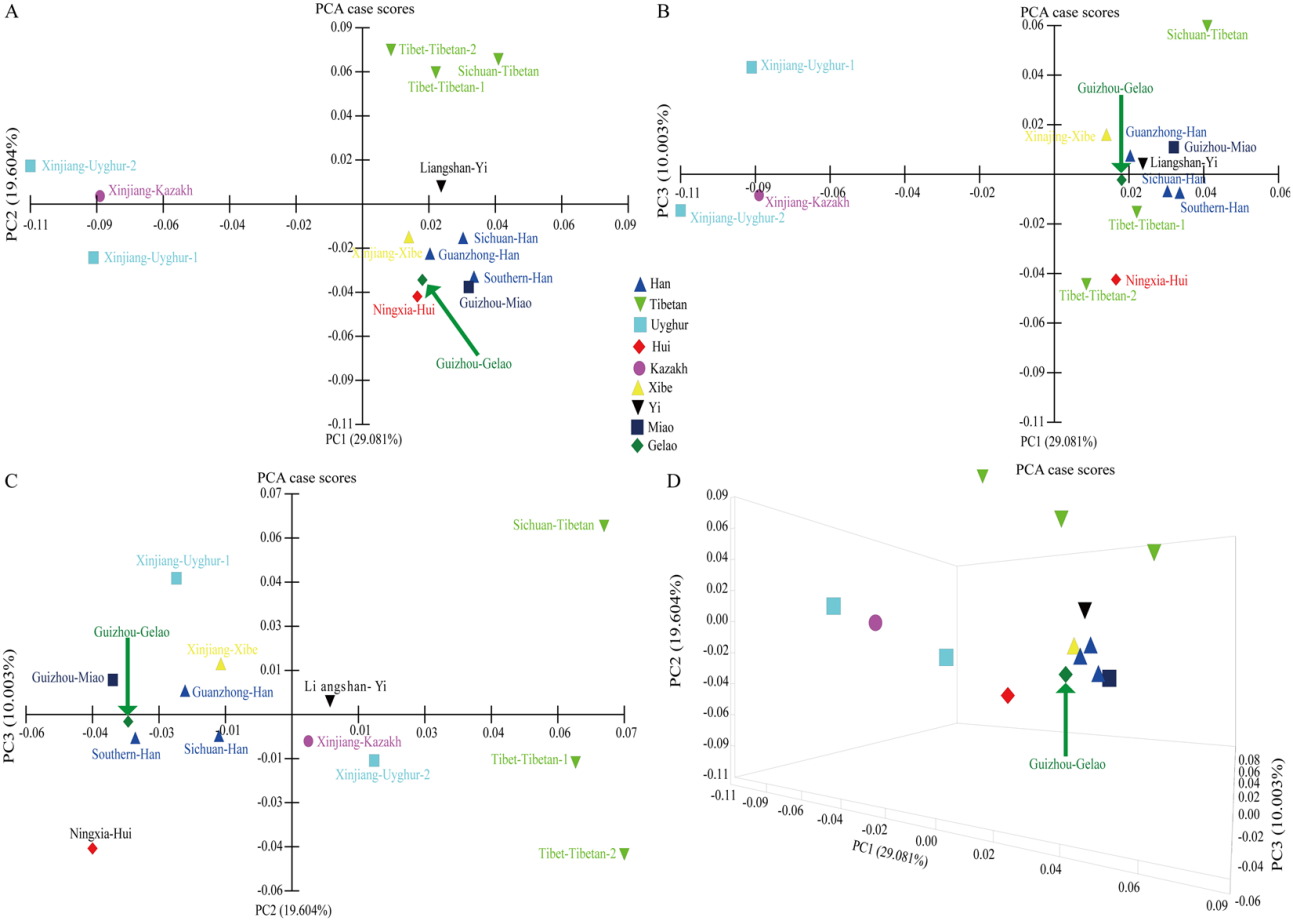
Principal component analysis (PCA) among 14 Chinese populations on the basis of genetic variants of 19 X-STRs. Scatter plots of 14 populations based on the first two PCAs reveal the genetic relationship between Guizhou Gelao and other 13 reference populations. (**A**) A series of other combinations of the first two PCAs show the population relationship: the combination of PCA1 and PCA3 (**B**), PCA2 and PCA3 (**C**), as well as the three-dimensional plots based on combinations of PCA1, PCA2 and PCA3 (**D**).

**Figure 3:**
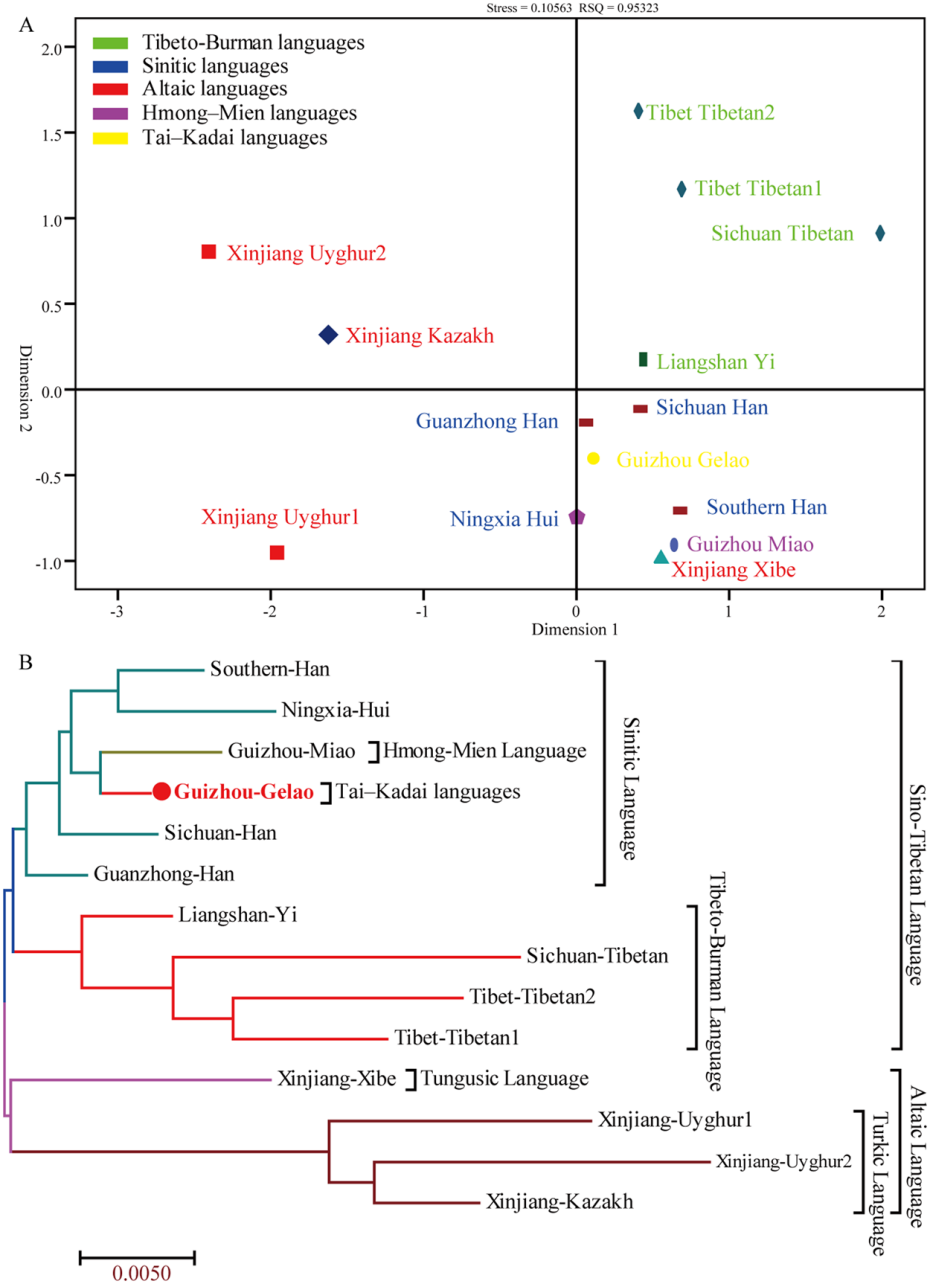
Genetic similarities and differences among 14 populations defined by ethnic origin and administrative divisions on the basis of Nei’s genetic distance matrix. (**A**) Multidimensional scaling plots among14 populations. (**B**) A phylogenetic tree on the basis of Neighbor-Joining algorithm was constructed among 14 populations.

We assessed the genetic relationships between the Guizhou Gelao and a panel of 13 nationwide populations^26–31,38–40^ consisting of 3,410 unrelated individuals genotyped with 19 X-STRs using Nei’s genetic distance, principal component analysis (PCA), multidimensional scaling analysis (MDS) and Neighbor-Joining (N-J) tree. The reference populations comprised Southern Han (n = 308)^30^, Tibet Tibetan2 (n = 213)^30^, Xinjiang Uyghur2 (n = 211)^30^, Ningxia Hui (n = 200)^30^, Tibet Tibetan1 (n = 270)^26^, Xinjiang Uygur1 (n = 220)^26^, Guanzhong Han (n = 474)^31^, Xinjiang Kazakh (n = 300)^39^, Xinjiang Xibe (n = 179)^40^, Liangshan Yi (n = 331)^27^, Sichuan Han (n = 201)^28^, Sichuan Tibetan (n = 235)^29^, and Guizhou Miao (n = 268)^60^. The first three principal components extracted 58.687% of total genetic variations (PC1: 29.081%, PC2: 19.604% and PC3: 10.003%). As showed in Fig. 2, PC1 can separate two Xinjiang Uyghur populations and one Kazakh population from others, and PC2 can differentiate three Tibetan populations from others. The third PC shows a separation of Ningxia Hui with other tested populations. PCA results on the basis of allele frequency distributions revealed that Guizhou Gelao is more closely related to Han Chinese populations, Miao and Xibe than to others. Pairwise comparisons between the studied Gelao and aforementioned 13 populations were subsequently estimated using the Nei’s genetic distances (Supplementary Table S10 and Fig. S6). A middle genetic heterogeneity (mean ± SD: 0.0262 ± 0.0110) among Chinese populations with the genetic distances spanning from 0.0070 (between Guanzhong Han and Guizhou Gelao) to 0.0519 (between Xinjiang Uyghur2 and Sichuan Tibetan) is observed. Guizhou Gelao is similarly related to Guanzhong Han (0.0070) and has a distant genetic relationship with Xinjiang Uyghur2 (0.0394), which is consistent with the population origin. Subsequently, we conducted the MDS based on the genetic distance matrix to further explore the genetic relationship and language affinity. As shown in Fig. 3, three Altaic-speaking populations are located in the second and third quadrants with the exception of Xinjiang Xibe located in the fourth quadrant. Four Tibeto-Burman-speaking populations are located in the first quadrant. However, Gelao, as one Tai-Kadai-speaking population, is located in the fourth quadrant and has high genetic affinity with Sinitic-speaking populations. One Hmong-Mien-speaking population of Guizhou Miao is positioned between Southern Han and Xinjiang Xibe. An N-J tree was constructed among these 14 populations belonged to four language families. We identified three main clusters: Altaic-speaking cluster, Tibeto-Burman-speaking cluster, and Sinitic-speaking cluster. Guizhou Gelao and Guizhou Miao form one branch and then grouped with Sinitic-speaking populations in the same cluster.

### Genetic relationships and population structures revealed by 11-overlapped STRs among 23 nationwide populations

**Figure 4:**
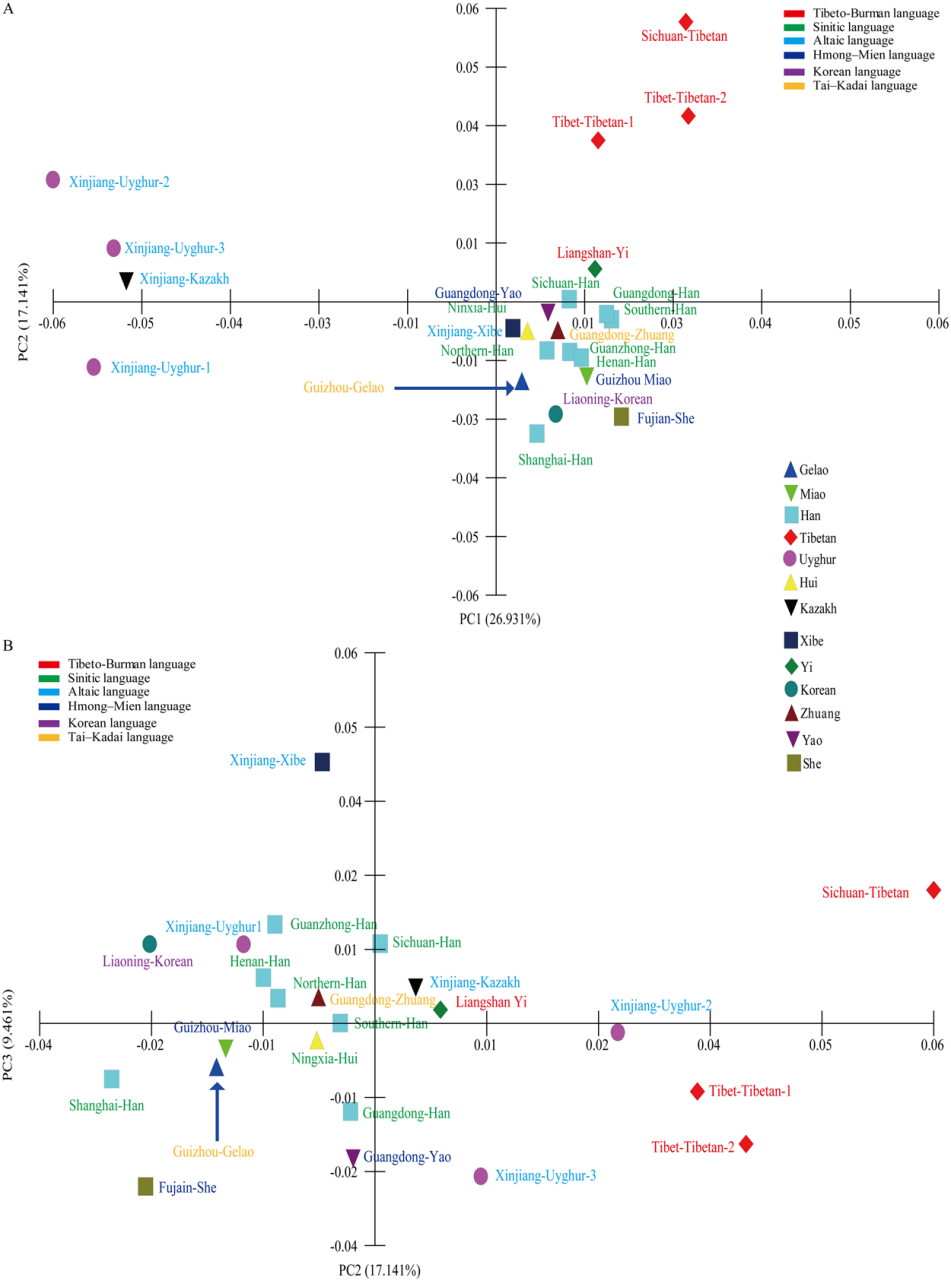
Principal component analysis (PCA) among 23 Chinese populations on the basis of genetic variations of 11 overlapping X-chromosomal STRs. (**A**) Genetic relationship revealed by the first two components (PCA1 and PCA2 coordinates). (**B**) Genetic differences and similarities among 24 populations are revealed by the combination of PCA1 and PCA3.

To glean further details of Chinese genetic structure, we combined our investigated genetic variations of Gelao with more previously published populations, including 22 reference groups^26–31,39–44,46–49^ from 12 diverse ethnicities and six language families, on the basis of 11 overlapped X-chromosomal genetic markers between the Investigator Argus X-12 QS Kit and AGCU X-19 amplification system. We first explored the genetic homogeneity and heterogeneity using PCA based on the allelic frequency distributions. We found that a total of 53.534% genetic variation was extracted from the total variance based on the first three components. As shown in Fig. 4, PC1 (26.931%) can distinguish Altaic-speaking populations with the exception of Xinjiang Xibe and PC2 (17.141%) separates Tibeto-Burman-speaking populations and PCA3 (9.461%) can successfully separate Xinjiang Xibe and Fujian She from others. The studied Gelao can be separated and keep a close relationship with other populations, such as Sinitic-speaking populations. Figure 4A on the basis of the combination of PC1 and PC2 shows one tight cluster consisting of eight Sinitic-speaking populations, three Hmong-Mien-speaking populations, one Korean-speaking population and two Tai-Kadai-speaking populations. Meanwhile, two separated Tibeto-Burman-speaking and Altaic-speaking clusters are located on the circumjacent regions. Guizhou Gelao is centrally located on the tight cluster. However, all populations are scattered in the two dimensional plots (PC2 and PC3) in Fig. 4B.

**Figure 5:**
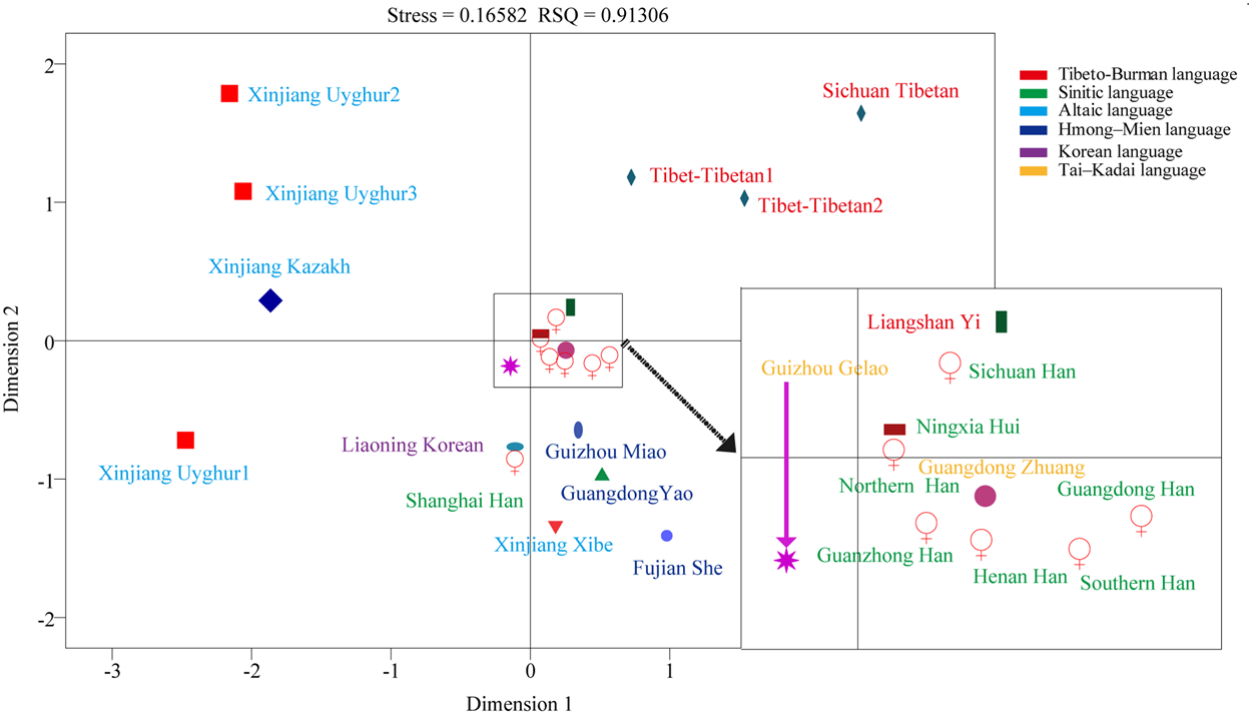
Multidimensional scaling plots revealing the genetic affinity among 23 populations belonging to six language families based on 11 overlapping X-chromosomal STRs.

**Figure 6:**
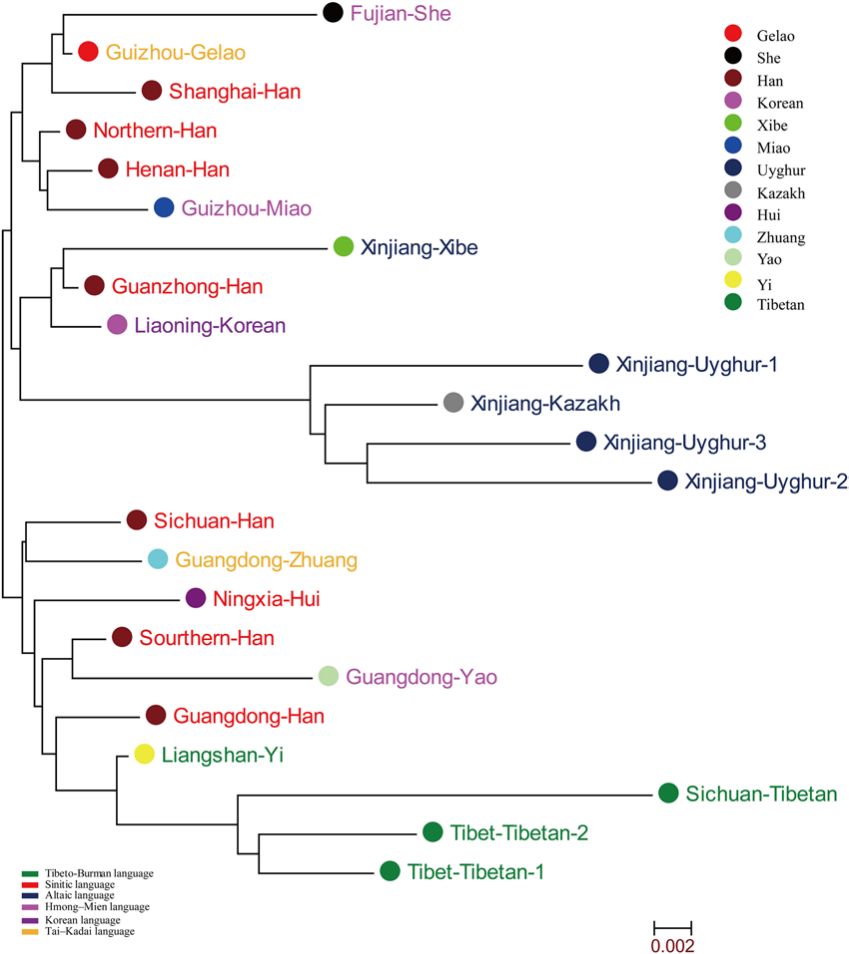
Neighbor-Joining tree constructed on the basis of Nei’s genetic distance matrix using the Mega 7.0 software to reconstruct the phylogenetic relationships between Guizhou Gelao and other 22 Chinese reference populations.

Pairwise Nei’s genetic distances between the Guizhou Gelao and other 22 nationwide reference populations^26–31,39–44,46–49^ are estimated and presented in Supplementary Table S11 and Fig. S7. The largest Nei’s genetic distance among 23 populations is observed between Sichuan Tibetan and Xinjiang Uyghur2 (0.0711) and the smallest is found between Northern Han and Henan Han (0.0037) with the mean ± standard deviation is 0.0262 ± 0.0141. Guizhou Gelao has a close genetic relationship with Northern Han (0.0054) and a distinct genetic relationship with Sichuan Tibetan (0.0422). Genetic relationships between Guizhou Gelao and reference populations were then explored using MDS plots. As shown in Fig. 5, Seven Sinitic-speaking populations, one Liangshan Yi, Guangdong Zhuang and Guizhou Gelao are centrally located on the MDS. Tibeto-Burman-speaking populations, Altaic-speaking and Hmong-Mien-speaking populations are respectively positioned in the first, second and third, fourth quadrants. Finally, we reconstructed phylogenetic relationships using the N-J tree. Three distinct clusters are obviously observed in Fig. 6: the upper group consists of three Han Chinese populations, Fujian She, Guizhou Gelao and Guizhou Miao. The middle group is made up of four Altaic-speaking populations (three Xinjiang Uyghurs and one Xinjiang Kazakh), Guanzhong Han, Liaoning Korean and Xinjiang Xibe. The lower group comprises four Tibeto-Burman-speaking populations (Liangshan Yi and three Tibetans), three Han Chinese populations, Ningxia Hui, Guangdong Zhuang and Guangdong Yao. Guizhou Gelao is first clustered with Fujian She, and then clustered together with Shanghai Han.

## Discussions

### Linkage and linkage disequilibrium

Forensic genetic workers are needed to illuminate the potential power (genetic polymorphisms and forensic parameters) in forensic application, in order to provide population-specific reference data for establishing a comprehensive database for a new PCR amplification system before its extensive use in forensic casework. Herein, Genotype data of 19 X-STRs included in AGCU X19 kit in 513 unrelated Chinese Gelao individuals is obtained. Before analyzing the forensic population frequency data, we evaluate the linkage disequilibrium. Linkage is the phenomenon that genetic markers are close together on a chromosome and can inherit as a unit during the meiosis phase of sexual reproduction. Linkage disequilibrium, also referred as allelic association, is non-random association of different alleles, which can be caused by linkage and specific population history, like population substructure, migration, non-random mating and genetic drift. In this study, linkage disequilibrium analyses were performed in both male and females. Most marker pairs which are of disequilibrium were observed within the linkage groups. Previous studies^49,55–59^ based on large scale pedigree and population genetic analyses revealed that the 19 X-STRs can be grouped into seven linkage groups (LG): LG1 comprises three loci located on X-chromosomal short arm^55,56^, LG2 is consisted of three genetic markers located on the centromere with low recombination rate^57^, LG3^49^, LG4^58^, LG5^59^, LG6^56^, LG7^49^ are located on the long arm. DNA Commission of the International Society for Forensic Genetics (ISFG) recently recommended that haplotype frequency should be considered to calculate the likelihood when linkage inheritance exists in the included forensic X-STRs^50^. Thus, statistical parameters of forensic interest based on both single locus and linkage groups are analyzed.

### Forensic efficiency

AGCU X-19 STR amplification system, co-amplification and fluorescent detection of the 19 X-STRs, was developed specifically to facilitate Chinese X-STR reference database establishment. To explore the power of this panel in forensic complex paternity testing and individual identification, we next comprehensively evaluate the forensic efficiency indexes and the genetic polymorphisms. A set of forensic parameters has been devised^51–54,61^, including GD, PIC, PE, PD_f_, PD_m_, and four mean paternity exclusion change indexes (MEC Krüger, MEC Kishida, MEC Desmarais and MEC Desmarais Duo). PIC and GD are serviceable in both autosomal and X-chromosomal markers, and GD is also appropriate for Y-chromosomal markers^16,61^. MEC Krüger is conceived for addressing the deficiency cases without the alleged father which replaced by the paternal grandmother using X-chromosomal markers and normal trios using autosomal markers^52^. MEC Kishida and MEC Desmarais are specially designed and suitable for trios with a daughter^53,54^, and MEC Desmarais Duo is valid for cases of father/daughter duos or mother/son duos on the basis of X-chromosomal markers^54^. In this study, the combined powers of the aforesaid six parameters in Chinese Gelao pooled population on the basis of single locus allele frequencies are respectively 0.99999999999985, 0.99999999999999999999973, 0.999999975715109, 0.999999999998337, 0.999999999998324, and 0.999999995577508. For haplotype analyses, the combined powers of discrimination and mean paternity exclusion chances are also estimated. The combined PD_m_ and PD_f_ are 0.999999999997095 and 0.99999999999999999999918, respectively, which are slightly smaller than efficiency calculated by allele frequency distributions. The cumulative mean paternity exclusion chances in trios are 0.999999999394923 (Krüger), 0.999999999991709 (Kishida), 0.999999999996492 (Desmarais), and which in duos is 0.999999999682643 (Desmarais). The combined MEC Kishida, and MEC Desmarais based on genetic polymorphisms in the single locus are larger than that on the basis of genetic variation of haplotype distributions of seven linkage groups. However, the higher combined indexes of MEC Krüger and MEC Desmarais Duo are observed according to the genetic polymorphisms of haplotype. Our findings combined with our previous investigations^27–29^ indicate that the 19 X-STRs are informative and polymorphic in Chinese Gelao population and this amplification system can efficiently complement the analyses of autosomal^13^, mitochondrial and Y-chromosomal STRs^16^, single nucleotide polymorphisms (SNPs)^62^, insertion/deletions (InDels)^63^ in the forensic applications, especially in some special and complicated kinship cases (deficiency kinships cases of paternal grandmother/granddaughter duos, mother-son duos, and full or half-sibling duos involving two females, as well as some specific incest cases).

### Population genetic relationship

China, located on the East Asia and comprising 56 ethnically/linguistically diverse ethnicities officially recognized by the People’s Republic of China and several unrecognized populations (such as Mosuo, Miyao), has been the genetic subject in the molecular anthropology, archaeology, population genetics and forensic genetics to shed light on the genetic diversity, origin, divergence, evolution, population migration and admixture of the eastern anatomically modern humans after migrating out of Africa around fifty millennium BC^32–35^. The detailed genetic structures of Chinese minority ethnicities with the exception of Uyghur and Tibetan^32,64^, particularly the Chinese Gelao, remains unresolved. We used two different datasets to investigate Chinese population structure. Significant genetic differences were identified between Turkic-speaking, Tibeto-Burman-speaking and other Chinese populations. Which are consisted with previous genetic studies^32,35,64^. Zhang *et al*. recently found the differentiated demographic histories of two Tibeto-Burman populations (Tibetan and Sherpa) and other east Asian populations, as well as revealed the high-altitude local adaptations (EPAS1and EGLN1)^64^. Feng *et al*. revealed four-way ancestries in modern Uyghurs (Turkic population): European and South Asian, East Asian and Siberian^32^. The specific genetic ancestry and population history, including high-altitude selection and admixture with surrounding populations, make a significantly different genetic architecture in Tibetans and Uyghurs.

Except for Turkic and Tibeto-Burman populations, other Chinese populations are homogenous groups as revealed in this study. Our comprehensive population genetic comparisons demonstrated that Gelao keeps the genetic affinity with this homogenous group, especially for Han Chinese and Guizhou Miao (geographically-neighboring population). Since the remarkable cluster structure was displayed by different methods between Gelao and these diverse ethnic groups from different linguistic family, including mainly Sinitic-speaking (Han, Hui), Hmong-Mien-speaking (Miao, Yao, She), and Tai-Kadai-speaking (Zhuang). Meanwhile, the closer genetic relationships between Gelao and others based on different methods and datasets are somewhat different: PCA revealed the Gelao shows close relationships mainly with Han, Miao and Xibe in Fig. 2, whereas with Han, Miao, Zhuang and Hui in Fig. 4; MDS revealed the closer genetic affinity between Gelao and Sinitic-speaking populations, Miao, and Xibe in Fig. 3, whereas and Sinitic-speaking populations, Zhuang, followed by Miao, Yao, Xibe and She in Fig. 5; N-J tree revealed Gelao grouped with Guizhou Miao fisrt in one branch and then clustered together with Sinitic-speaking populations in Fig. 3, whereas Gelao first clustered with Fujian She, and then clustered together with Shanghai Han, Guizhou Miao and two other Han populations in Fig. 6. Three software programs (PCA, MDS and phylogenetic tree) are the most well-known and widely used methods for examining the general patterns of population genetic relationships. Although, overall consensus was showed among the Gelao and other homogeneous populations, the completely same results about the closer genetic relationships between the Gelao and others cannot be obtained by using distinct descriptive methods, like the conclusions revealed by formal tests of Admixturetools^65^ or TreeMix^66^. Which is also consisted with previous studies based on the Y-chromosomal, autosomal genetic markers^13,14,67,68^. Totally, our results based on the X-chromosomal markers demonstrated genetic differentiations among Turkic, Tibeto-Burman and other admixture groups (homogeneous populations, including Gelao). These patterns of genetic variation and structure are caused by the migration^34,35^, nature selection^64^, admixture^32^ and religious and cultural diffusion^13,17,34^.

As a typical example of the apparent genetic affinity between the Gelao and all compared Han populations derived from distinct administrative regions as shown by all three phylogenetic methods, it can also be explained as a mixed cluster pattern: an obvious ethnical cluster of different Han populations coupled with a probable geographical cluster of the Gelao ethnicity and local Han majority, since they have a long history of living and intermarriage with each other in the same northern part of Guizhou Province^36,37,69,70^. Additionally, Guizhou Miao is another minority group in Guizhou Province and geographically close to Guizhou Gelao^60^. The close genetic relationships of Guizhou Miao and Guizhou Gelao are displayed more explicitly and steadily than others (except for Han Chinese) based on all the tested methods, To better understand the origin and migration of Gelao ethnicity and dissect the fine-scale genetic structures and relationships with complex surrounding or related populations, additional genome and population analyses based on higher resolution genetic marker sets, such as high-density SNP chip and whole-genome sequencing data, are needed.

## Conclusions

Tightly linked X-STR markers play an important role in forensic complex kinship cases or deficiency case identifications. In this study, we genotyped 19 X-STRs in 513 unrelated Chinese Gelao individuals to investigate the forensic characteristics, and combined with 13 previously studied nationwide populations based on the genetic variations of 19 X-STRs as well as 22 reference populations on the basis of 11 overlapping X-STRs to explore the Chinese population genetic relationships along ethnic, geographical and linguistic divisions. All 19 X-STRs are in accordance with the HWE. Forensic parameters are estimated according to both allele and haplotype frequency distributions. Locus of DXS10135 and linkage group of DXS10148-DXS10135-DXS8378 are the most informative and polymorphic genetic markers in Chinese Gelao population. The high combined power discrimination and mean paternity exclusion chance are achieved based on genetic variations of both 19 X-STRs and 7 linkage groups with minor differences, indicating that this panel could complement the applications of autosomal, Y-chromosomal and mitochondrial markers in forensic deficiency cases. This study also provides haplotype database for likelihood estimation of kinship identification in Guizhou Gelao. Additionally, our PCA, MDS and phylogenetic relationship reconstruction, which are based on two sets of genetic markers from a large of Chinese populations, are concordant in revealing the genetic distinctions among Tibeto-Burman-speaking populations, Altaic-speaking populations and other Chinese language family populations. Besides, Guizhou Gelao as a Tai-Kadai-speaking population, has the closer genetic relationship with Han Chinese and geographically close Guizhou Miao. Further genetic studies based on the whole-genome studies of modern or archaic samples in East Asia are needed due to the existing uncertainty of genetic relationships among Chinese populations.

## Methods and Materials

### Compliance with ethical standards and sample collections

This study was performed with the approval of the Ethics Committee of the Zunyi Medical University and followed the guidelines published by Center of Forensic Expertise, Affiliated Hospital of Zunyi Medical University. Each voluntary participant has signed the written informed consent after being informed of the aim of the study. A total of 513 human blood samples (265 females and 248 males) were collected from unrelated healthy Gelao individuals residing in the Zunyi City in Guizhou Province, southwest China. Samples from individuals whose parents and paternal grandparents belonged to the Gelao ethnolinguistic group and had non-consanguineous marriages within three generations.

### DNA extraction and quantification

Genomic DNA was extracted and isolated using the salting-out method. Quantification analysis of DNA template was carried out using the Quantifiler Human DNA Quantification Kit (Thermo Fisher Scientific) on the basis of manufacturer’s instruction on the 7500 Real-Time PCR System (Thermo Fisher Scientific). All DNA sample was diluted to 1 ng/μl and preserved in the −20 °C until the following amplification.

### Amplification and genotyping

19 X-chromosomal STR loci (DXS7132, DXS10079, DXS6789, DXS101, DXS10103, DXS10101, HPRTB, DXS10075, DXS10074, DXS10135, DXS7423, DXS10148, DXS10159, DXS6809, DXS7424, DXS8378, DXS10164, DXS10162, and DXS10134) included in the AGCU X19 STR Kit (AGCU ScienTech Incorporation, Wuxi, Jiangsu, China) were co-amplified according to the manufacturer’s protocol on a GeneAmp PCR System 9700 Thermal Cycler (Thermo Fisher Scientific, MA, USA) using the 25 μL reaction volume which contains reaction mix (10 μL), A-Taq DNA polymerase (0.5 μL), primers (5 μL), template DNA (2 μL), and sdH_2_O (7.5 μL). PCR cycling was employed as 95 °C for 2 min, followed by 10 cycles of 94 °C for 30 s, 60 °C for 1 min, 65 °C for 1 min, and then followed by 20 cycles of 94 °C for 30 s, 59 °C for 1 min, 72 °C for 1 min, and a final extension at 60 °C for 30 min and 4 °C preservation. PCR products were isolated and detected using the capillary electrophoresis (36 cm capillary array) with POP-4 polymer in the Applied 3500 Genetic Analyzer (Thermo Fisher Scientific, MA, USA) which used 5 s at 3 kV for sample injection and 15 kV for 1500 s at 60 °C for electrophoresis. Allele allocation of gene fragment was conducted using the GeneMapper ID-X v.1.4 software (Thermo Fisher Scientific) in combination with the set of bins and panels, the allelic ladder, and AGCU Marker Size-500 following the manufacturer’s instruction.

### Analytical method

We calculated the allele frequencies of 19 X-STRs in the Gelao males, females and pooled population using the modified PowerStat V1.2 spreadsheet (Promega, Madison WI, USA). Haplotype distributions and corresponding haplotype frequencies of seven linkage groups were estimated by the direct counting. Forensic statistical parameters polymorphism information content (PIC), power of exclusion (PE), paternity index (PI), power of discrimination in female (PD_f_) and male (PD_m_) and mean paternity exclusion chance (MEC) for trios cases introduced respectively by Krüger *et al*.^52^ (MEC Krüger), Kishida *et al*.^53^ (MEC Kishida), and Desmarais *et al*.^54^ (MEC Desmarais) and for duos cases introduced by Desmarais *et al*.^54^ (MEC Desmarais Duo) were evaluated using the online tool provided by the ChrX-STR.org 2.0 database (http://www.chrx-str.org/). Gene diversity (GD) and haplotype diversity (HD) were estimated using Nei’s formula^51^:

1 $$GD/HD=\frac{N}{N-1}(1-\sum {{P}_{i}}^{2}),$$

and match probability (MP) was evaluated using the following formula:

2 $${\rm{MP}}=\sum {{P}_{i}}^{2},$$

where *N* and *P*_*i*_ respectively denote the population size and *i*th allele frequency or haplotype frequency. The gender differentiation (Fst and corresponding p values), Hardy-Weinberg equilibrium (HWE) in females, Linkage disequilibrium (LD) in males and females were calculated using the Arlequin software (version 3.5.2)^71^. Finally, we used the newly developed software StatsX (Statistics for X-STR) v2.0^72^ to examine and validate our analysis results.

To dissect the genetic heterogeneity and homogeneity between the studied Gelao population and other nationwide reference populations along ethnic, linguistic and administrative divisions, we first integrated our data with 13 previously investigated populations genotyped by 19 X-STRs and then combined our data with 22 reference populations on the basis of the overlapped 11 X-STRs (DXS7132, DXS10079, DXS10074, DXS10103, HPRTB, DXS10101, DXS10134, DXS10148, DXS10135, DXS8378, and DXS7423). The first set of reference groups: Southern Han^30^, Tibet Tibetan2^30^, Xinjiang Uyghur2^30^, Ningxia Hui^30^, Tibet Tibetan1^26^, Xinjiang Uygur1^26^, Guanzhong Han^31^, Xinjiang Kazakh^39^, Xinjiang Xibe^40^, Liangshan Yi^27^, Sichuan Han^28^, Sichuan Tibetan^29^, Guizhou Miao^60^. The second set of reference groups: Guizhou Miao, Southern Han^30^, Tibet Tibetan2^30^, Xinjiang Uyghur2^30^, Ningxia Hui^30^, Tibet Tibetan1^26^, Xinjiang Uygur1^26^, Guanzhong Han^31^, Xinjiang Kazakh^39^, Xinjiang Xibe^40^, Liangshan Yi^27^, Sichuan Han^28^, Sichuan Tibetan^29^, Northern Han^41^, Guangdong Han^48^, Shanghai Han^49^, Henan Han^44^, Liaoning Korean^42^, Guangdong Zhuang^46^, Guangdong Yao^47^, Fujian She^43^, Xinjiang Uyghur3^38^. The pairwise Nei’s genetic distances between Guizhou Gelao and aforementioned reference populations were estimated using the PHYLIP ver. 3.5 packages^73^. Principal component analyses of the two sets of populations on the basis of allele frequency distributions were performed using the Multivariate Statistical Package (MVSP) version 3.22 software^74^. Multidimensional scaling analyses and phylogenetic relationships on the basis of the Neighbor-Joining algorithm were conducted respectively using IBM SPSS Statistics version 21 (SPSS, Chicago, IL, USA)^75^ and Molecular Evolutionary Genetics Analysis Version 7.0 (Mega 7.0)^76^.

### Quality control

Most of our experiments in this study (DNA quantification, amplification, capillary electrophoresis) were performed in the Department of Forensic Genetics, West China School of Basic Medical Sciences & Forensic Medicine, Sichuan University. This laboratory has passed the China National Accreditation Service for Conformity Assessment (CNAS) and the accreditation of ISO 17025. Besides, this department has taken part in international cooperation of Y-STR genotype quality control. Our experimental procedure was strictly following the guidelines and recommendations of this laboratory. The 9947A cell line and ddH_2_O were intended for use as the controls in each batch of genotype.

## Electronic supplementary material

Supplementary Figures S1-S6 and Tables S1-S11.
