# Supplementary material for: Genetic structure and polymorphisms of Gelao ethnicity residing in southwest china revealed by X-chromosomal genetic markers
Source: Sci Rep. 2018 Oct 1;8:14585. doi: 10.1038/s41598-018-32945-7 (PMC6167355; doi:10.1038/s41598-018-32945-7)
Supplement: Supplementary file 1 — Supplementary Figures S1-S6 and Tables S1-S11. [file 41598_2018_32945_MOESM1_ESM.pdf]

Supplementary Figures S1-S6 and Tables S1-S11

Genetic structure and polymorphisms of Gelao ethnicity residing in southwest china revealed by X-chromosomal genetic markers

Pengyu Chen[1,2,+], Guanglin He[3,+], Xing Zou[3], Mengge Wang[3], Haibo Luo[3], Limei Yu[4], Xijie Hu[4], Mijia Xia[5], Hongyan Gao[1,2], Jian Yu[1,2], Yiping Hou[3,*],Yanyan Han[6,*]

[1]Center of Forensic Expertise, Affiliated Hospital of Zunyi Medical University, Zunyi, Guizhou, China
[2]Department of Forensic Medicine, Zunyi Medical University, Zunyi, Guizhou, China
[3]Institute of Forensic Medicine, West China School of Basic Medical Sciences & Forensic Medicine, Sichuan University, Chengdu, Sichuan, China
[4]Key Laboratory of Cell Engineering in Guizhou Province, Zunyi, Guizhou, China
[5]Judicial Authentication Institution, First People's Hospital of Zunyi City, Zunyi, Guizhou, China
[6]Department of Nutrition and Food Hygiene, School of Public Health, Zunyi Medical University, Zunyi, Guizhou, China

+ These authors contributed equally to this work.

* Correspondence and requests for materials should be addressed to
Yanyan Han (hanyanyan1984@126.com)
Yiping Hou (profhou@yahoo.cn)

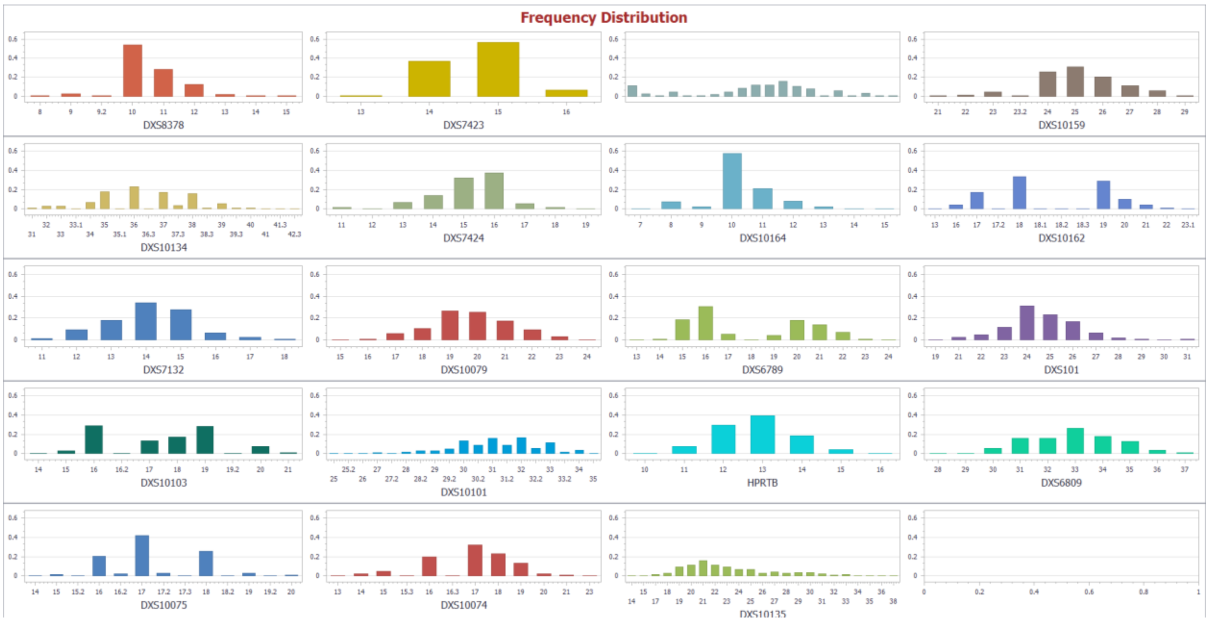

Supplementary Figure S1. The allele of 19 X-chromosomal STRs and corresponding allele frequency distributions in the Guizhou Gelao female population.

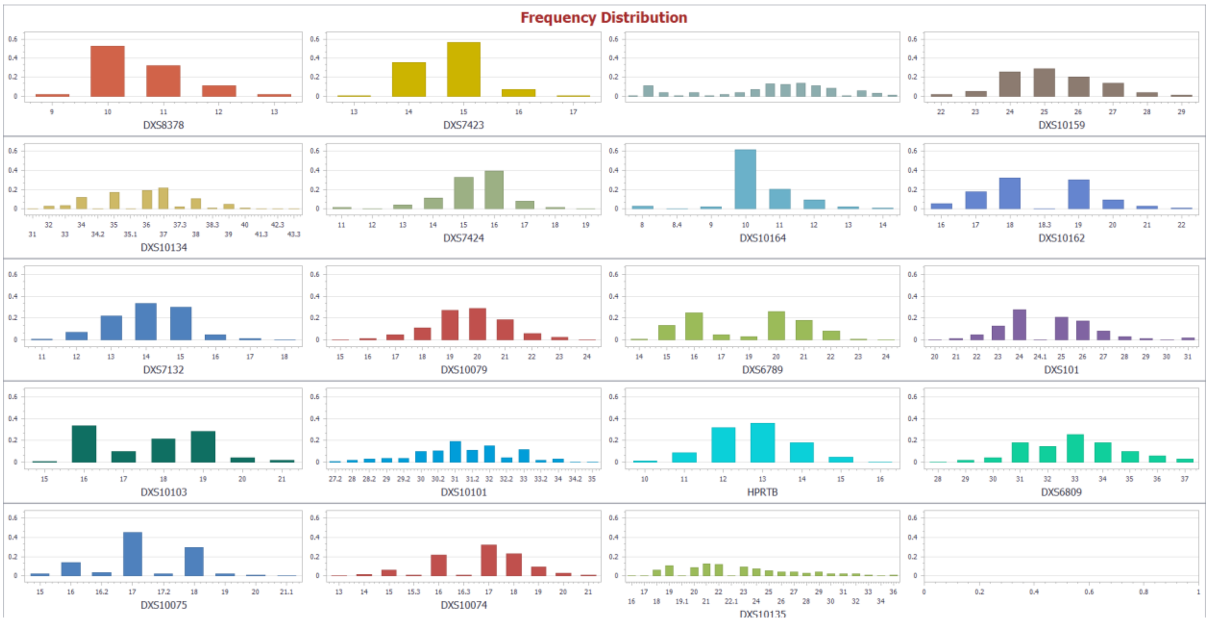

Supplementary Figure S2. The allele of 19 X-chromosomal STRs and corresponding allele frequency distributions in the Guizhou Gelao male population.

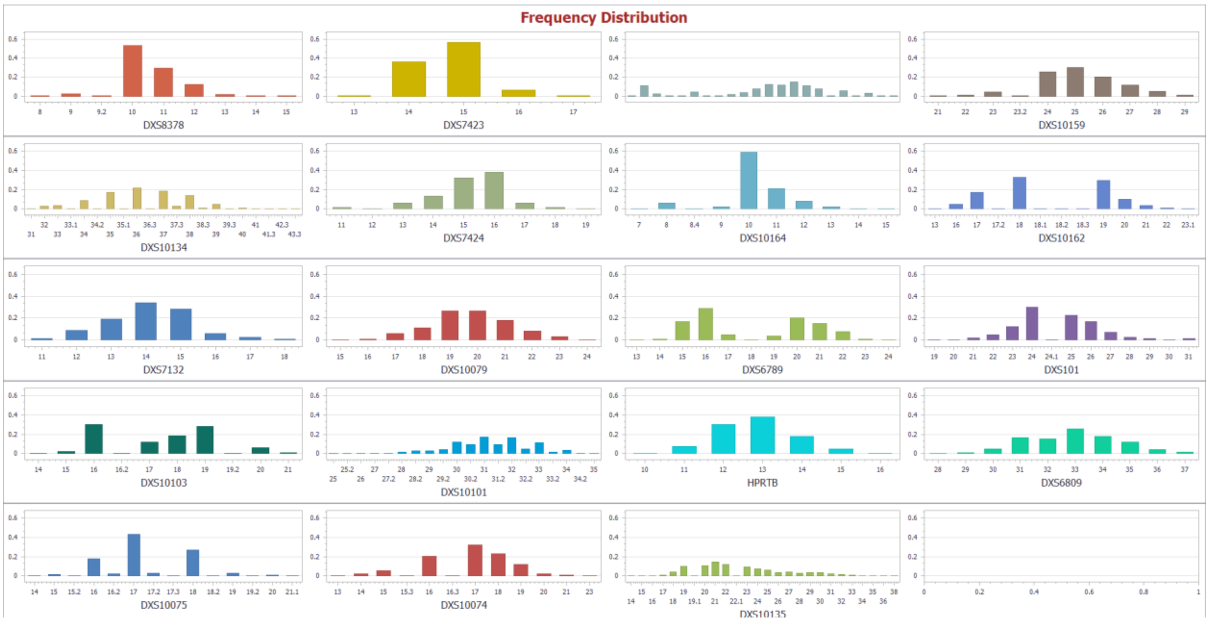

Supplementary Figure S3. The allele of 19 X-chromosomal STRs and corresponding allele frequency distributions in the Guizhou Gelao pooled males and females.

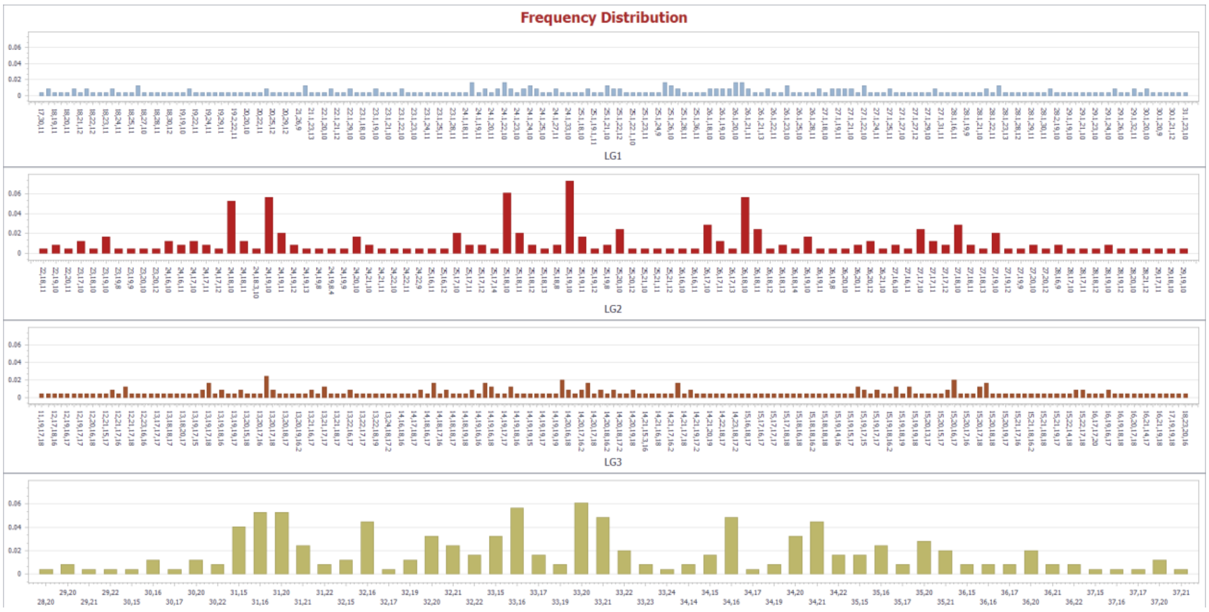

Supplementary Figure S4. The haplotype of four linkage groups consisting of 12 X-chromosomal STRs and corresponding haplotype frequency distributions in the Guizhou Gelao male population. LG1: DXS10148-DXS10135-DXS8378; LG2: DXS10159-DXS10162-DXS10164; LG3: DXS7132-DXS10079-DXS10074-DXS10075; LG4: DXS6809-DXS6789.

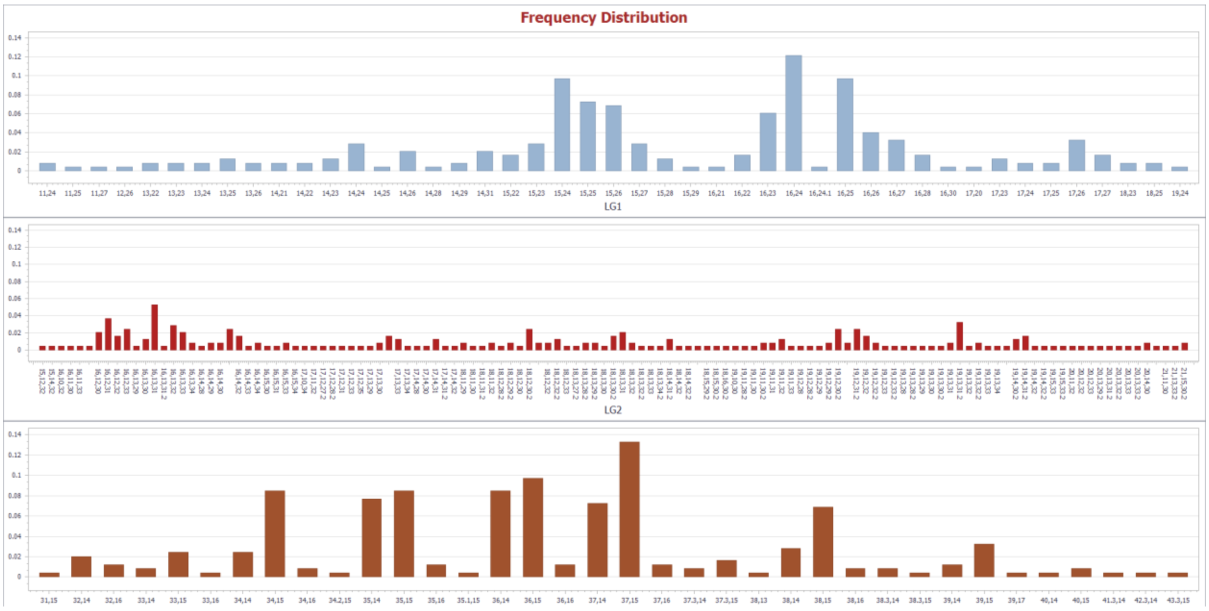

Supplementary Figure S5. The haplotype of three linkage groups consisting of 19 X-chromosomal STRs and corresponding haplotype frequency distributions in the Guizhou Gelao male population. LG1: DXS7424-DXS101; LG2: DXS10103-HPRTB-DXS10101; LG3: DXS10134-DXS7423.

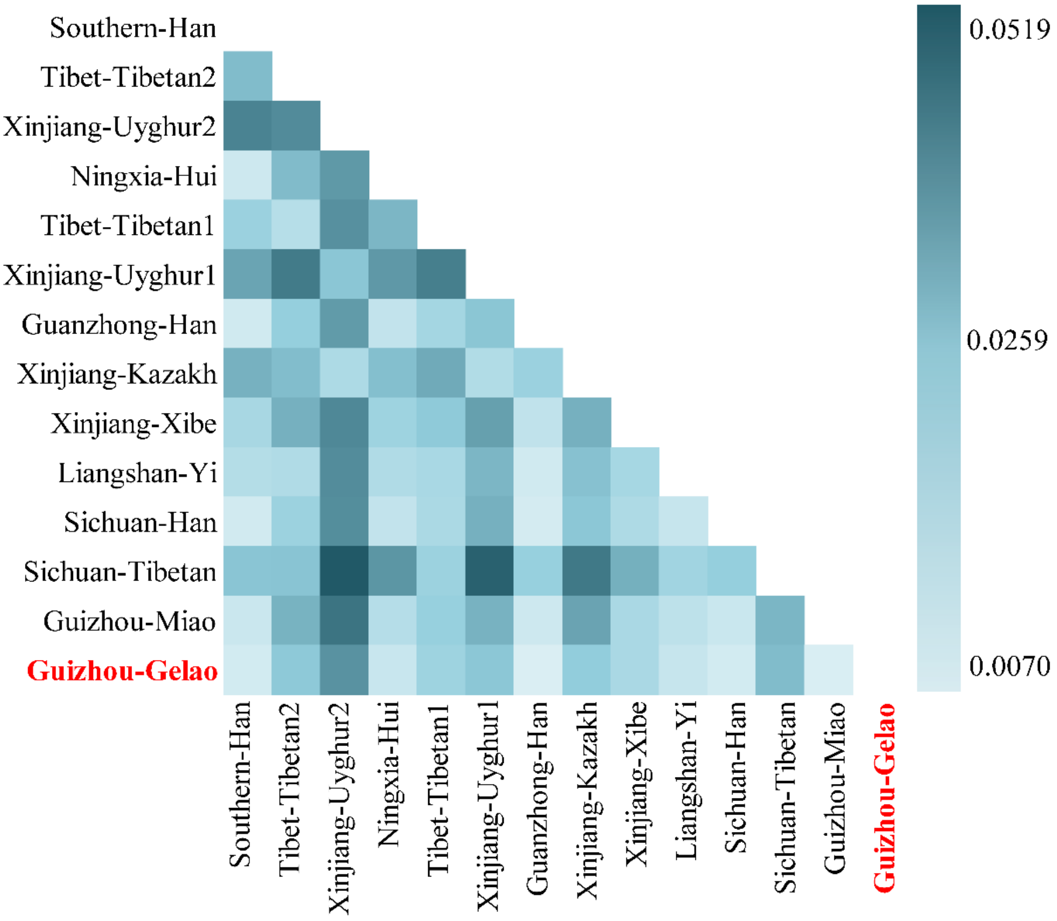

Supplementary Figure S6. The hot plots of pairwise genetic distances among 14 Chinese populations.

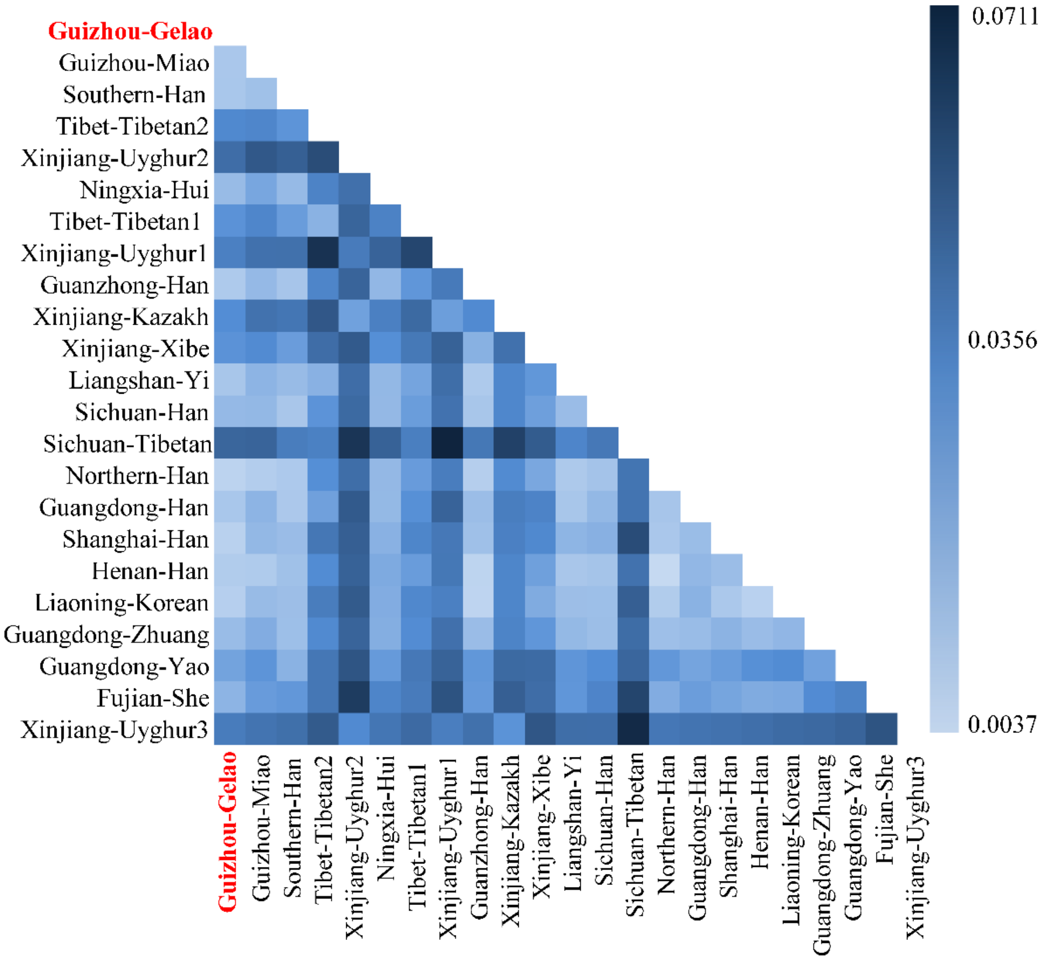

Supplementary Figure S7. The hot plots of pairwise genetic distances between Guizhou Gelao and 22 reference populations on the basis of 11 overlapped X-STRs.

Supplementary Table S1. The p values of the pairwise linkage disequilibrium in all pairs of 19 X-STR loci in 265 female individuals residing in Guizhou Province

Loci	DXS8378	DXS7423	DXS10148	DXS10159	DXS10134	DXS7424	DXS10164	DXS10162	DXS7132	DXS10079	DXS6789	DXS101	DXS10103	DXS10101	HPRTB	DXS6809	DXS10075	DXS10074	DXS10135
DXS8378																			
DXS7423	0.0952																		
DXS10148	0.9027	0.1552																	
DXS10159	0.8636	0.5817	0.8904																
DXS10134	0.0307	0.0894	0.1214	0.7905															
DXS7424	0.3079	0.8986	0.2034	0.7634	0.6744														
DXS10164	0.9653	0.4574	0.2464	0.4929	0.2466	0.8492													
DXS10162	0.0896	0.2047	0.5211	0.1371	0.5429	0.7163	0.7068												
DXS7132	0.4068	0.9025	0.7405	0.1399	0.9395	0.0979	0.0984	0.6524											
DXS10079	0.6970	0.4785	0.1968	0.7222	0.4961	0.2018	0.1333	0.8081	0.3788										
DXS6789	0.1433	0.6174	0.6091	0.6923	0.2917	0.2902	0.4407	0.9419	0.1983	0.4104									
DXS101	0.7488	0.4122	0.7522	0.6846	0.8797	0.1046	0.9614	0.2020	0.4936	0.3350	0.7649								
DXS10103	0.2899	0.0983	0.4501	0.7861	0.1717	0.1929	0.7743	0.5542	0.7287	0.8726	0.5910	0.0812							
DXS10101	0.4001	0.3956	0.1956	0.1321	0.4122	0.6804	0.9863	0.3938	0.1128	0.5032	0.9184	0.6808	0.0000						
HPRTB	0.1218	0.3884	0.6311	0.8327	0.0395	0.6737	0.1962	0.0617	0.0841	0.0614	0.3005	0.0895	0.2389	0.5204					
DXS6809	0.6217	0.6189	0.2727	0.8482	0.0927	0.8482	0.6494	0.3435	0.4345	0.0308	0.3371	0.5602	0.4921	0.1764	0.0127				
DXS10075	0.4041	0.3575	0.3295	0.3606	0.5017	0.9712	0.1909	0.1844	0.3838	0.0459	0.0269	0.6212	0.3813	0.2838	0.7276	0.7542			
DXS10074	0.5416	0.5297	0.2473	0.1635	0.8257	0.1471	0.2319	0.8146	0.6843	0.0680	0.1342	0.9091	0.9399	0.2420	0.3451	0.2803	0.7022		
DXS10135	0.5851	0.2265	0.1099	0.2941	0.8000	0.3855	0.2511	0.0578	0.2307	0.9041	0.2706	0.4542	0.7668	0.2085	0.0647	0.0253	0.3227	0.2519	

Supplementary Table S2. The p values of the pairwise linkage disequilibrium in all pairs of 19 X-STR loci in 248 male individuals residing in Guizhou Province

Loci	DXS8378	DXS7423	DXS10148	DXS10159	DXS10134	DXS7424	DXS10164	DXS10162	DXS7132	DXS10079	DXS6789	DXS101	DXS10103	DXS10101	HPRTB	DXS6809	DXS10075	DXS10074	DXS10135
DXS8378																			
DXS7423	0.1040																		
DXS10148	0.3185	0.9205																	
DXS10159	0.3722	0.6226	0.0605																
DXS10134	0.9250	0.0278	0.0000	0.0110															
DXS7424	0.6425	0.6968	0.7878	0.4059	0.7756														
DXS10164	0.0000	0.6240	0.1012	0.2358	0.6753	0.6958													
DXS10162	0.1891	0.9303	0.3306	0.0206	0.1380	0.4451	0.0239												
DXS7132	0.3273	0.7790	0.2356	0.4900	0.3820	0.6582	0.1206	0.8535											
DXS10079	0.6296	0.0530	0.2340	0.3717	1.0000	0.7229	0.2696	0.7142	0.0542										
DXS6789	0.6361	0.3677	0.9893	0.1290	0.8714	0.1665	0.9137	0.3768	0.6993	0.4057									
DXS101	0.5638	0.0916	0.2667	0.3909	0.2665	0.0000	0.5143	0.2375	0.7896	0.7518	0.1968								
DXS10103	0.7164	0.7551	0.5103	0.1445	0.0796	0.6339	0.6800	0.1172	0.7357	0.9061	0.3642	0.7136							
DXS10101	0.9494	0.5584	0.7759	0.6079	0.0477	0.0588	0.8674	0.3061	0.0908	0.2063	0.1984	0.2091	0.0000						
HPRTB	0.4731	0.7887	0.1464	0.8332	0.8021	0.3081	0.2267	0.7954	0.2763	0.5900	0.1724	0.1611	0.0932	0.1530					
DXS6809	0.5757	0.3376	0.8376	0.5849	0.9861	0.0392	0.4551	0.7339	0.5089	0.0858	0.7642	0.0532	0.0208	0.5112	0.9994				
DXS10075	0.4724	0.6774	0.3036	0.2166	0.2631	0.3889	0.1475	0.7581	0.7844	0.2585	0.4699	0.7482	0.0192	0.3970	0.2525	0.3866			
DXS10074	0.1481	0.9187	0.3941	0.2252	0.1137	0.8045	0.3993	0.8310	0.3817	0.3328	0.9485	0.7349	0.7663	0.7911	0.7527	0.1219	0.2783		
DXS10135	0.1921	0.0722	0.2717	0.2139	0.1602	0.9589	0.1163	0.0993	0.9581	0.0697	0.3967	0.0894	0.8951	0.0069	0.7174	0.0032	0.0004	0.6019	

Supplementary Table S3. Allele frequencies of 19 X-chromosomal STRs in 265 Zunyi Gelao female individuals

Allele	DXS8378	DXS7423	DXS10148	DXS10159	DXS10134	DXS7424	DXS10164	DXS10162	DXS7132	DXS10079	DXS6789	DXS101	DXS10103	DXS10101	HPRTB	DXS6809	DXS10075	DXS10074	DXS10135
7							0.0019												
8	0.0019						0.0774												
9	0.0245						0.0208												
9.2	0.0019																		
10	0.5377						0.5736								0.0019				
11	0.283					0.017	0.2132		0.0113						0.0736				
12	0.1264					0.0057	0.0792		0.0943						0.2962				
13	0.0208	0.0038				0.0698	0.0245	0.0019	0.1811		0.0019				0.3943			0.0019	
14	0.0019	0.3679				0.1415	0.0057		0.3415		0.0075		0.0038		0.183		0.0019	0.0226	0.0019
15	0.0019	0.5642				0.3226	0.0038		0.2755	0.0019	0.1868		0.0283		0.0453		0.0132	0.0509	0.0019
15.2																	0.0019		
15.3																		0.0019	
16		0.0642				0.3736		0.0434	0.0642	0.0075	0.3075		0.2906		0.0057		0.2019	0.1981	
16.2													0.0019				0.0208		
16.3																		0.0057	
17						0.0528		0.1698	0.0264	0.0604	0.0509		0.1321				0.4208	0.3208	0.0132

17.2						0.0019									0.0321		
17.3															0.0038		
18	0.1113			0.0151		0.334	0.0057	0.1075	0.0038		0.1736				0.2585	0.2321	0.0321
18.1						0.0019											
18.2						0.0019									0.0038		
18.3						0.0019											
19	0.0226			0.0019		0.2906		0.2642	0.0434	0.0019	0.283				0.0302	0.134	0.0925
19.1	0.0019																
19.2											0.0019				0.0019		
20	0.0453					0.1019		0.2547	0.1774		0.0736				0.0094	0.0226	0.1132
20.1	0.0057																
21	0.0019	0.0019				0.0396		0.1755	0.1377	0.0245	0.0113					0.0075	0.1566
21.1	0.017																
22		0.0132				0.0094		0.0943	0.0698	0.0453							0.1151
22.1	0.0415																
23		0.0453						0.0321	0.0094	0.117						0.0019	0.0925
23.1	0.0811					0.0019											
23.2		0.0019															
24		0.2566						0.0019	0.0038	0.3113							0.0698
24.1	0.117																
25		0.3075								0.2302		0.0019					0.0679
25.1	0.1189																
25.2												0.0019					
26		0.2038								0.1679		0.0019					0.0321
26.1	0.1566																

27		0.1075		0.0642	0.0094		0.0434
27.1	0.1057						
27.2					0.0019		
28		0.0547		0.0189	0.0132	0.0057	0.0321
28.1	0.0755						
28.2	0.0038				0.0302		
29		0.0075		0.0094	0.0302	0.0057	0.0358
29.1	0.0547						
29.2	0.0019				0.0472		
30				0.0038	0.1321	0.0566	0.0377
30.1	0.0321						
30.2	0.0019				0.0849		
31			0.0075	0.0057	0.1585	0.1623	0.0208
31.1	0.0038						
31.2					0.0906		
32			0.0302		0.1679	0.1604	0.0113
32.2					0.0528		
33			0.0321		0.1151	0.2623	0.0151
33.1			0.0019				
33.2					0.0189		
34			0.0698		0.0358	0.1755	0.0057
35			0.1755		0.0057	0.1264	0.0057
35.1			0.0019				
36			0.2283			0.034	0.0019
36.3			0.0019				

37	0.1717		0.0113	
37.3	0.034			
38	0.1585			0.0019
38.3	0.0075			
39	0.0528			
39.3	0.0075			
40	0.0075			
41	0.0019			
41.3	0.0057			
42.3	0.0038			

Supplementary Table S4. Allele frequencies of 19 X-chromosomal STRs in 248 Guizhou Gelao male individuals

Allele	DXS8378	DXS7423	DXS10148	DXS10159	DXS10134	DXS7424	DXS10164	DXS10162	DXS7132	DXS10079	DXS6789	DXS101	DXS10103	DXS10101	HPRTB	DXS6809	DXS10075	DXS10074	DXS10135
7																			
8							0.028												
8.4							0.004												
9	0.02						0.024												
10	0.528						0.617								0.012				
11	0.319					0.016	0.202		0.008						0.085				
12	0.113					0.004	0.093		0.073						0.315				
13	0.02	0.004				0.044	0.024		0.218						0.359			0.004	
14		0.355				0.113	0.008		0.335		0.008				0.177			0.016	
15		0.569				0.327			0.302	0.004	0.133		0.008		0.048		0.02	0.061	
15.3																		0.008	
16		0.069				0.395		0.057	0.048	0.012	0.25		0.335		0.004		0.137	0.218	0.004
16.2																	0.036		
16.3																		0.008	
17		0.004	0.004			0.081		0.177	0.012	0.048	0.048		0.101				0.452	0.323	0.004
17.2																	0.024		
18			0.109			0.016		0.319	0.004	0.109			0.214				0.294	0.23	0.065
18.3								0.004											
19			0.036			0.004		0.302		0.27	0.028		0.282				0.024	0.097	0.109
19.1																			0.004
19.2			0.004																
20			0.04					0.097		0.286	0.262	0.004	0.044				0.008	0.028	0.089

21	0.004			0.032	0.186	0.177	0.012	0.016				0.008	0.125
21.1	0.016										0.004		
22		0.016		0.012	0.057	0.081	0.048						0.121
22.1	0.036												0.004
23		0.048			0.024	0.008	0.129						0.097
23.1	0.069												
24		0.254			0.004	0.004	0.274						0.077
24.1	0.133						0.004						
25		0.29					0.206						0.057
25.1	0.121												
26		0.202					0.173						0.04
26.1	0.137												
27		0.137					0.081						0.044
27.1	0.113												
27.2									0.008				
28		0.04					0.032		0.016	0.004			0.032
28.1	0.081												
28.2	0.004								0.028				
29		0.012					0.012		0.036	0.016			0.044
29.1	0.057												
29.2									0.036				
30							0.004		0.097	0.04			0.024
30.1	0.028												
30.2									0.105				
31			0.004				0.02		0.194	0.177			0.02

31.1	0.008				
31.2			0.109		
32		0.032	0.153	0.145	0.02
32.2			0.044		
33		0.036	0.117	0.254	0.008
33.2			0.016		
34		0.117	0.032	0.177	0.004
34.2		0.004	0.004		
35		0.173	0.004	0.097	
35.1		0.004			
36		0.194		0.061	0.008
37		0.218		0.028	
37.3		0.024			
38		0.109			
38.3		0.012			
39		0.048			
40		0.012			
41.3		0.004			
42.3		0.004			
43.3		0.004			

Supplementary Table S5. The Fst and corresponding p values between male and female Gelao population

Loci	pairwise Fst	p values
DXS101	-0.0021	0.81962 ±0.0037
DXS6789	0.0015	0.20097 ±0.0038
DXS6809	-0.0023	0.87437 ±0.0035
DXS7132	-0.0019	0.70092 ±0.0048
DXS7423	-0.0038	0.97129 ±0.0017
DXS7424	-0.0021	0.74686 ±0.0042
DXS8378	-0.0026	0.76339 ±0.0043
DXS10074	-0.0025	0.85754 ±0.0034
DXS10075	0.0012	0.24760 ±0.0043
DXS10079	-0.0021	0.78933 ±0.0036
DXS10101	-0.0017	0.83140 ±0.0037
DXS10103	0.0000	0.38155 ±0.0046
DXS10134	0.0009	0.26898 ±0.0043
DXS10135	-0.0020	0.93832 ±0.0025
DXS10148	-0.0032	0.99941 ±0.0002
DXS10159	-0.0030	0.94070 ±0.0022
DXS10162	-0.0034	0.98535 ±0.0012
DXS10164	0.0003	0.33274 ±0.0049
HPRTB	-0.0027	0.85298 ±0.0039

Supplementary Table S6. Allele frequencies of 19 X-chromosomal STRs in the Guizhou Gelao population (265 females and 248 males)

Allele	DXS8378	DXS7423	DXS10148	DXS10159	DXS10134	DXS7424	DXS10164	DXS10162	DXS7132	DXS10079	DXS6789	DXS101	DXS10103	DXS10101	HPRTB	DXS6809	DXS10075	DXS10074	DXS10135
7							0.0013												
8	0.0013						0.0617												
8.4							0.0013												
9	0.0231						0.0219												
9.2	0.0013																		
10	0.5347						0.5874								0.0051				
11	0.2943					0.0167	0.2095		0.0103						0.0771				
12	0.1221					0.0051	0.0835		0.0874						0.3021				
13	0.0206	0.0039				0.0617	0.0244	0.0013	0.1928		0.0013				0.383			0.0026	
14	0.0013	0.3638				0.1324	0.0064		0.3393		0.0077		0.0026		0.1812		0.0013	0.0206	0.0013
15	0.0013	0.5656				0.3239	0.0026		0.2841	0.0026	0.1697		0.0219		0.0463		0.0154	0.054	0.0013
15.2																	0.0013		
15.3																		0.0039	
16		0.0656				0.3805		0.0476	0.0591	0.009	0.2892		0.3046		0.0051		0.1812	0.2044	0.0013
16.2													0.0013				0.0257		
16.3																		0.0064	

17	0.0013	0.0013		0.0617	0.1722	0.0219	0.0566	0.0501		0.1221		0.4306	0.3213	0.0103
17.2					0.0013							0.0296		
17.3												0.0026		
18		0.1105		0.0154	0.329	0.0051	0.108	0.0026		0.1864		0.2699	0.2314	0.0424
18.1					0.0013									
18.2					0.0013							0.0026		
18.3					0.0026									
19		0.027		0.0026	0.2943		0.2661	0.0386	0.0013	0.2828		0.0283	0.1221	0.0977
19.1		0.0013												0.0013
19.2		0.0013								0.0013		0.0013		
20		0.0437			0.1003		0.2648	0.2044	0.0013	0.0643		0.009	0.0244	0.1054
20.1		0.0039												
21		0.0026	0.0013		0.0373		0.1787	0.1504	0.0206	0.0129			0.0077	0.1465
21.1		0.0167										0.0013		
22			0.0141		0.0103		0.0823	0.0733	0.0463					0.117
22.1		0.0398												0.0013
23			0.0463				0.0296	0.009	0.1208				0.0013	0.0938
23.1		0.0771			0.0013									
23.2			0.0013											
24			0.2558				0.0026	0.0039	0.2995					0.072
24.1		0.1221							0.0013					
25			0.3021						0.2224		0.0013			0.0643
25.1		0.1195												
25.2											0.0013			
26			0.2031						0.1697		0.0013			0.0347

26.1	0.1504						
27		0.117		0.0694	0.0064		0.0437
27.1	0.108						
27.2					0.0039		
28		0.0501		0.0231	0.0141	0.0051	0.0321
28.1	0.0771						
28.2	0.0039				0.0296		
29		0.009		0.0103	0.0321	0.009	0.0386
29.1	0.0553						
29.2	0.0013				0.0437		
30				0.0039	0.1208	0.0514	0.0334
30.1	0.0308						
30.2	0.0013				0.0913		
31			0.0064	0.0103	0.1697	0.1671	0.0206
31.1	0.0051						
31.2					0.0964		
32			0.0308		0.1632	0.1555	0.0141
32.2					0.0501		
33			0.0334		0.1157	0.2596	0.0129
33.1			0.0013				
33.2					0.018		
34			0.0848		0.0347	0.1761	0.0051
34.2			0.0013		0.0013		
35			0.1748		0.0051	0.117	0.0039
35.1			0.0026				

36	0.2172			0.0424	0.0039
36.3	0.0013				
37	0.1864			0.0167	
37.3	0.0308				
38	0.1427				0.0013
38.3	0.009				
39	0.0514				
39.3	0.0051				
40	0.009				
41	0.0013				
41.3	0.0051				
42.3	0.0039				
43.3	0.0013				

Supplementary Table S7. Forensic parameters of 19 X-STRs in Guizhou Gelao female individuals

Forensic Parameters	N alleles	GD	PIC	PDM	PDF	MEC Kruger	MEC Kishida	MEC Desmarais	MEC Desmarais duo
DXS8378	9	0.6149	0.5548	0.6138	0.7919	0.3582	0.5548	0.5548	0.4077
DXS7423	4	0.5432	0.4523	0.5422	0.7005	0.2580	0.4524	0.4523	0.3140
DXS10148	20	0.9048	0.8951	0.9031	0.9826	0.8041	0.8953	0.8951	0.8173
DXS10159	10	0.7827	0.7485	0.7812	0.9194	0.5769	0.7484	0.7485	0.6184
DXS10134	19	0.8530	0.8346	0.8514	0.9611	0.7049	0.8346	0.8346	0.7298
DXS7424	9	0.7295	0.6849	0.7281	0.8829	0.4991	0.6849	0.6849	0.5453
DXS10164	9	0.6133	0.5722	0.6122	0.8096	0.3882	0.5723	0.5722	0.4238
DXS10162	13	0.7627	0.7247	0.7612	0.9065	0.5470	0.7248	0.7247	0.5903
DXS7132	8	0.7622	0.7241	0.7608	0.9061	0.5454	0.7241	0.7241	0.5894
DXS10079	10	0.8109	0.7832	0.8093	0.9376	0.6260	0.7832	0.7832	0.6614
DXS6789	12	0.8121	0.7863	0.8106	0.9398	0.6323	0.7861	0.7863	0.6655
DXS101	12	0.8025	0.7745	0.8010	0.9340	0.6164	0.7746	0.7745	0.6510
DXS10103	10	0.7830	0.7486	0.7815	0.9193	0.5763	0.7487	0.7486	0.6182
DXS10101	19	0.8935	0.8821	0.8918	0.9786	0.7823	0.8822	0.8821	0.7978
HPRTB	7	0.7172	0.6681	0.7158	0.8715	0.4740	0.6681	0.6681	0.5255
DXS6809	10	0.8294	0.8055	0.8278	0.9480	0.6579	0.8057	0.8055	0.6897
DXS10075	13	0.7140	0.6676	0.7127	0.8724	0.4777	0.6678	0.6676	0.5254
DXS10074	12	0.7838	0.7503	0.7823	0.9206	0.5796	0.7503	0.7503	0.6205
DXS10135	23	0.9158	0.9079	0.9141	0.9864	0.8272	0.9081	0.9079	0.8375

N, Number of alleles; GD, gene diversity; PIC, polymorphism information content; PDF, power of discrimination in females; PDM, power of discrimination in males; MEC Krüger, mean paternity exclusion chance for autosomal STR markers in trios and complex kinship cases; MEC Kishida, mean paternity exclusion chance for X-chromosomal markers in trios involving daughters; MEC Desmarais, mean paternity exclusion chance for X-chromosomal markers in trios involving daughters (Desmarais version); MEC Desmarais Duo, Mean paternity exclusion chance for X-chromosomal markers in father/daughter duos.

Supplementary Table S8. Forensic parameters of 19 X-chromosomal STRs in Gelao male individuals

Forensic Parameters	N	HD	PIC	PDM	PDF	MEC Krüger	MEC Kishida	MEC Desmarais	MEC Desmarais duo
DXS10148	18	0.90808	0.89655	0.90442	0.98299	0.80622	0.89644	0.89655	0.81948
DXS10135	22	0.92292	0.91349	0.91919	0.98776	0.83664	0.91370	0.91349	0.84621
DXS8378	5	0.60845	0.53906	0.60600	0.77782	0.34011	0.53906	0.53906	0.39313
DXS10159	8	0.79059	0.75548	0.78740	0.92288	0.58519	0.75536	0.75548	0.62660
DXS10162	8	0.76496	0.72491	0.76187	0.90633	0.54614	0.72491	0.72491	0.59041
DXS10164	8	0.57045	0.52821	0.56815	0.77357	0.34694	0.52809	0.52821	0.38011
DXS7132	8	0.74430	0.69756	0.74130	0.88934	0.50838	0.69756	0.69756	0.55855
DXS10079	10	0.79566	0.76277	0.79246	0.92724	0.59790	0.76289	0.76277	0.63609
DXS10074	11	0.78459	0.74962	0.78143	0.92042	0.58003	0.74962	0.74962	0.62004
DXS10075	9	0.69039	0.63948	0.68761	0.85429	0.44895	0.63960	0.63948	0.49550
DXS6809	10	0.83912	0.81544	0.83573	0.95273	0.67385	0.81533	0.81544	0.70330
DXS6789	10	0.81311	0.78365	0.80984	0.93765	0.62632	0.78365	0.78365	0.66190
DXS7424	9	0.71828	0.67036	0.71538	0.87397	0.48330	0.67024	0.67036	0.52939
DXS101	13	0.82854	0.80332	0.82520	0.94756	0.65712	0.80321	0.80332	0.68776
DXS10103	7	0.75320	0.70883	0.75016	0.89625	0.52241	0.70895	0.70883	0.57118
HPRTB	7	0.73409	0.68626	0.73113	0.88284	0.49600	0.68626	0.68626	0.54590
DXS10101	16	0.88974	0.87568	0.88615	0.97657	0.77153	0.87557	0.87568	0.78833
DXS10134	17	0.85735	0.83744	0.85389	0.96219	0.70901	0.83721	0.83744	0.73367
DXS7423	5	0.54841	0.46059	0.54620	0.70845	0.26603	0.46034	0.46059	0.32112

N, Number of alleles; HD, gene diversity; PIC, polymorphism information content; PDF, power of discrimination in females; PDM, power of discrimination in males; MEC Krüger, mean paternity exclusion chance for autosomal STR markers in trios and complex kinship cases; MEC Kishida, mean paternity exclusion chance for X-chromosomal markers in trios involving daughters; MEC Desmarais, mean paternity exclusion chance for X-chromosomal markers in trios involving daughters (Desmarais version); MEC Desmarais Duo, Mean paternity exclusion chance for X-chromosomal markers in father/daughter duos.

Supplementary Table S9. The haplotype and corresponding haplotype frequencies of Seven linkage groups in Gelao male population (n = 248)

LG1	Count	Frequency	LG2	Count	Frequency	LG3	Count	Frequency	LG4	Count	Frequency	LG5	Count	Frequency	LG6	Count	Frequency	LG7	Count	Frequency
17 30 11	1	0.0040	22 18 11	1	0.0040	11 19 17 18	1	0.0040	28 20	1	0.0040	11 24	2	0.0081	15 12 32	1	0.0040	31 15	1	0.0040
18 19 10	2	0.0081	22 19 10	2	0.0081	11 22 18 18	1	0.0040	29 20	2	0.0081	11 25	1	0.0040	15 14 32	1	0.0040	32 14	5	0.0202
18 19 11	1	0.0040	22 20 11	1	0.0040	12 17 18 16	1	0.0040	29 21	1	0.0040	11 27	1	0.0040	16 10 32	1	0.0040	32 16	3	0.0121
18 19 13	1	0.0040	23 17 10	3	0.0121	12 19 15 17	1	0.0040	29 22	1	0.0040	12 26	1	0.0040	16 11 30	1	0.0040	33 14	2	0.0081
18 20 11	1	0.0040	23 18 10	1	0.0040	12 19 16 17	1	0.0040	30 15	1	0.0040	13 22	2	0.0081	16 11 33	1	0.0040	33 15	6	0.0242
18 21 10	2	0.0081	23 19 10	4	0.0161	12 19 16 18	1	0.0040	30 16	3	0.0121	13 23	2	0.0081	16 12 29	1	0.0040	33 16	1	0.0040
18 21 12	1	0.0040	23 19 8	1	0.0040	12 19 17 17	1	0.0040	30 17	1	0.0040	13 24	2	0.0081	16 12 30	5	0.0202	34 14	6	0.0242
18 22 10	2	0.0081	23 19 9	1	0.0040	12 19 17 18	1	0.0040	30 20	3	0.0121	13 25	3	0.0121	16 12 31	9	0.0363	34 15	21	0.0847
18 22 12	1	0.0040	23 20 10	1	0.0040	12 20 16 18	1	0.0040	30 22	2	0.0081	13 26	2	0.0081	16 12 32	4	0.0161	34 16	2	0.0081
18 23 10	1	0.0040	23 20 12	1	0.0040	12 20 18 17	1	0.0040	31 15	10	0.0403	14 21	2	0.0081	16 12 33	6	0.0242	34.2 15	1	0.0040
18 23 11	1	0.0040	24 16 10	3	0.0121	12 21 15 17	1	0.0040	31 16	13	0.0524	14 22	2	0.0081	16 13 29	1	0.0040	35 14	19	0.0766
18 24 10	2	0.0081	24 16 11	2	0.0081	12 21 16 17	2	0.0081	31 20	13	0.0524	14 23	3	0.0121	16 13 30	3	0.0121	35 15	21	0.0847
18 24 11	1	0.0040	24 17 10	3	0.0121	12 21 17 16	1	0.0040	31 21	6	0.0242	14 24	7	0.0282	16 13 31	13	0.0524	35 16	3	0.0121
18 24 12	1	0.0040	24 17 11	2	0.0081	12 21 17 17	3	0.0121	31 22	2	0.0081	14 25	1	0.0040	16 13 31.2	1	0.0040	35.1 15	1	0.0040
18 25 11	1	0.0040	24 17 12	1	0.0040	12 21 17 18	1	0.0040	32 15	3	0.0121	14 26	5	0.0202	16 13 32	7	0.0282	36 14	21	0.0847
18 26 10	3	0.0121	24 18 10	13	0.0524	12 21 18 17.2	1	0.0040	32 16	11	0.0444	14 28	1	0.0040	16 13 33	5	0.0202	36 15	24	0.0968
18 27 10	1	0.0040	24 18 11	3	0.0121	12 23 16 16	1	0.0040	32 17	1	0.0040	14 29	2	0.0081	16 13 34	2	0.0081	36 16	3	0.0121
18 28 10	1	0.0040	24 18.3 10	1	0.0040	13 17 15 17	1	0.0040	32 19	3	0.0121	14 31	5	0.0202	16 14 28	1	0.0040	37 14	18	0.0726
18 28 11	1	0.0040	24 19 10	14	0.0565	13 17 17 17	1	0.0040	32 20	8	0.0323	15 22	4	0.0161	16 14 29	2	0.0081	37 15	33	0.1331
18 29 10	1	0.0040	24 19 11	5	0.0202	13 18 16 17	1	0.0040	32 21	6	0.0242	15 23	7	0.0282	16 14 30	2	0.0081	37 16	3	0.0121
18 30 12	1	0.0040	24 19 12	2	0.0081	13 18 18 17	1	0.0040	32 22	4	0.0161	15 24	24	0.0968	16 14 31	6	0.0242	37.3 14	2	0.0081
18 32 10	1	0.0040	24 19 13	1	0.0040	13 18 18 18	1	0.0040	33 15	8	0.0323	15 25	18	0.0726	16 14 32	4	0.0161	37.3 15	4	0.0161
19 19 10	1	0.0040	24 19 8	1	0.0040	13 18 20 17	1	0.0040	33 16	14	0.0565	15 26	17	0.0685	16 14 33	1	0.0040	38 13	1	0.0040
19 19 11	2	0.0081	24 19 8.4	1	0.0040	13 19 15.3 16	1	0.0040	33 17	4	0.0161	15 27	7	0.0282	16 14 34	2	0.0081	38 14	7	0.0282
19 22 11	1	0.0040	24 19 9	1	0.0040	13 19 17 15	1	0.0040	33 19	2	0.0081	15 28	3	0.0121	16 15 30	1	0.0040	38 15	17	0.0685
19 24 10	1	0.0040	24 20 10	4	0.0161	13 19 17 17	2	0.0081	33 20	15	0.0605	15 29	1	0.0040	16 15 31	1	0.0040	38 16	2	0.0081
19 24 11	1	0.0040	24 21 10	2	0.0081	13 19 17 18	4	0.0161	33 21	12	0.0484	16 21	1	0.0040	16 15 33	2	0.0081	38.3 14	2	0.0081
19 27 10	1	0.0040	24 21 11	1	0.0040	13 19 17 19	1	0.0040	33 22	5	0.0202	16 22	4	0.0161	16 15 34	1	0.0040	38.3 15	1	0.0040
19 29 11	1	0.0040	24 22 10	1	0.0040	13 19 18 16	2	0.0081	33 23	2	0.0081	16 23	15	0.0605	17 10 34	1	0.0040	39 14	3	0.0121
19 30 11	1	0.0040	24 22 11	1	0.0040	13 19 18 17	1	0.0040	33 24	1	0.0040	16 24	30	0.1210	17 11 32	1	0.0040	39 15	8	0.0323

19.2 22 11	1	0.0040	24 22 9	1	0.0040	13 19 19 17	1	0.0040	34 14	2	0.0081	16 24.1	1	0.0040	17 12 27.2	1	0.0040	39 17	1	0.0040
20 18 12	1	0.0040	25 16 11	1	0.0040	13 20 15 17	2	0.0081	34 15	4	0.0161	16 25	24	0.0968	17 12 28.2	1	0.0040	40 14	1	0.0040
20 20 10	1	0.0040	25 16 12	1	0.0040	13 20 15 18	1	0.0040	34 16	12	0.0484	16 26	10	0.0403	17 12 31	1	0.0040	40 15	2	0.0081
20 22 10	1	0.0040	25 17 10	5	0.0202	13 20 16 17	1	0.0040	34 17	1	0.0040	16 27	8	0.0323	17 12 33	1	0.0040	41.3 14	1	0.0040
20 22 11	1	0.0040	25 17 11	2	0.0081	13 20 17 16	1	0.0040	34 19	2	0.0081	16 28	4	0.0161	17 12 35	1	0.0040	42.3 14	1	0.0040
20 23 10	2	0.0081	25 17 12	2	0.0081	13 20 17 17	6	0.0242	34 20	8	0.0323	16 30	1	0.0040	17 13 29	1	0.0040	43.3 15	1	0.0040
20 25 12	1	0.0040	25 17 14	1	0.0040	13 20 17 18	2	0.0081	34 21	11	0.0444	17 20	1	0.0040	17 13 30	2	0.0081			
20 29 11	1	0.0040	25 18 10	15	0.0605	13 20 18 16	1	0.0040	34 22	4	0.0161	17 23	3	0.0121	17 13 31	4	0.0161			
20 29 12	1	0.0040	25 18 11	5	0.0202	13 20 18 17	1	0.0040	35 15	4	0.0161	17 24	2	0.0081	17 13 33	3	0.0121			
20 31 11	1	0.0040	25 18 12	2	0.0081	13 20 18 18	1	0.0040	35 16	6	0.0242	17 25	2	0.0081	17 13 34	1	0.0040			
21 26 9	1	0.0040	25 18 13	1	0.0040	13 20 19 16.2	1	0.0040	35 17	2	0.0081	17 26	8	0.0323	17 14 28	1	0.0040			
21.1 20 10	3	0.0121	25 18 8	2	0.0081	13 20 21 18	1	0.0040	35 20	7	0.0282	17 27	4	0.0161	17 14 30	1	0.0040			
21.1 23 13	1	0.0040	25 19 10	18	0.0726	13 21 16 17	2	0.0081	35 21	5	0.0202	18 23	2	0.0081	17 14 31	3	0.0121			
22.1 19 10	1	0.0040	25 19 11	4	0.0161	13 21 16 18	1	0.0040	36 15	2	0.0081	18 25	2	0.0081	17 14 31.2	1	0.0040			
22.1 20 10	1	0.0040	25 19 12	1	0.0040	13 21 17 17	3	0.0121	36 16	2	0.0081	19 24	1	0.0040	17 14 32	1	0.0040			
22.1 21 10	2	0.0081	25 19 8	2	0.0081	13 21 17 18	1	0.0040	36 17	2	0.0081				18 11 29	2	0.0081			
22.1 21 12	1	0.0040	25 20 10	6	0.0242	13 21 18 17	1	0.0040	36 20	5	0.0202				18 11 30	1	0.0040			
22.1 23 10	1	0.0040	25 20 12	1	0.0040	13 21 18 18	1	0.0040	36 21	2	0.0081				18 11 31.2	1	0.0040			
22.1 29 10	2	0.0081	25 21 10	1	0.0040	13 22 16 17	2	0.0081	36 22	2	0.0081				18 11 32	2	0.0081			
22.1 31 10	1	0.0040	25 21 11	1	0.0040	13 22 17 16	1	0.0040	37 15	1	0.0040				18 12 28.2	1	0.0040			
23.1 18 10	1	0.0040	25 21 12	1	0.0040	13 22 17 17	1	0.0040	37 16	1	0.0040				18 12 29.2	2	0.0081			
23.1 18 11	1	0.0040	26 16 10	1	0.0040	13 22 18 17	1	0.0040	37 17	1	0.0040				18 12 30	1	0.0040			
23.1 19 10	2	0.0081	26 16 11	1	0.0040	13 22 18 19	1	0.0040	37 20	3	0.0121				18 12 30.2	6	0.0242			
23.1 20 12	1	0.0040	26 17 10	7	0.0282	13 23 17 16	1	0.0040	37 21	1	0.0040				18 12 31.2	2	0.0081			
23.1 21 10	1	0.0040	26 17 11	3	0.0121	13 24 18 17.2	1	0.0040							18 12 32	2	0.0081			
23.1 21 12	1	0.0040	26 17 13	1	0.0040	14 16 17 16	1	0.0040							18 12 32.2	3	0.0121			
23.1 22 10	2	0.0081	26 18 10	14	0.0565	14 16 18 16	1	0.0040							18 12 33	1	0.0040			
23.1 22 11	1	0.0040	26 18 11	6	0.0242	14 17 18 16	1	0.0040							18 13 27.2	1	0.0040			
23.1 23 10	1	0.0040	26 18 12	1	0.0040	14 17 18 17	1	0.0040							18 13 28.2	2	0.0081			
23.1 23 12	1	0.0040	26 18 13	2	0.0081	14 18 15 18	2	0.0081							18 13 29.2	2	0.0081			
23.1 24 11	1	0.0040	26 18 14	1	0.0040	14 18 16 17	1	0.0040							18 13 30	1	0.0040			
23.1 25 10	1	0.0040	26 19 10	4	0.0161	14 18 16 18	4	0.0161							18 13 30.2	4	0.0161			

23.1 25 11	1	0.0040	26 19 11	1	0.0040	14 18 17 16	1	0.0040	18 13 31	5	0.0202
23.1 27 11	1	0.0040	26 19 8	1	0.0040	14 18 18 16	2	0.0081	18 13 32	2	0.0081
23.1 28 11	1	0.0040	26 20 10	1	0.0040	14 18 18 17	1	0.0040	18 13 32.2	1	0.0040
24.1 18 10	1	0.0040	26 20 11	2	0.0081	14 18 18 18	1	0.0040	18 13 33	1	0.0040
24.1 18 11	1	0.0040	26 20 12	3	0.0121	14 18 19 18	1	0.0040	18 13 34.2	1	0.0040
24.1 19 10	4	0.0161	26 21 10	1	0.0040	14 18 20 18	2	0.0081	18 14 31.2	3	0.0121
24.1 19 11	1	0.0040	27 16 10	2	0.0081	14 19 16 16	1	0.0040	18 14 32	1	0.0040
24.1 20 10	2	0.0081	27 16 11	1	0.0040	14 19 16 17	4	0.0161	18 14 32.2	1	0.0040
24.1 20 11	1	0.0040	27 17 10	6	0.0242	14 19 16 18	3	0.0121	18 14 33	1	0.0040
24.1 21 12	2	0.0081	27 17 11	3	0.0121	14 19 16.3 17	1	0.0040	18 15 29.2	1	0.0040
24.1 22 10	4	0.0161	27 17 12	2	0.0081	14 19 17 17	1	0.0040	18 15 30.2	1	0.0040
24.1 22 11	2	0.0081	27 18 10	7	0.0282	14 19 17 18	3	0.0121	18 16 30.2	1	0.0040
24.1 23 10	1	0.0040	27 18 11	2	0.0081	14 19 18 16	1	0.0040	19 10 30	1	0.0040
24.1 23 11	2	0.0081	27 18 13	1	0.0040	14 19 18 17	1	0.0040	19 11 28.2	1	0.0040
24.1 24 10	3	0.0121	27 19 10	5	0.0202	14 19 19 15	1	0.0040	19 11 30	1	0.0040
24.1 24 11	2	0.0081	27 19 12	1	0.0040	14 19 19 16	1	0.0040	19 11 30.2	2	0.0081
24.1 25 10	1	0.0040	27 19 9	1	0.0040	14 19 19 17	1	0.0040	19 11 31	2	0.0081
24.1 25 12	1	0.0040	27 20 10	2	0.0081	14 19 19 18	1	0.0040	19 11 32	3	0.0121
24.1 27 11	2	0.0081	27 20 12	1	0.0040	14 19 19 19	1	0.0040	19 11 33	1	0.0040
24.1 30 13	1	0.0040	28 16 9	2	0.0081	14 20 16 17	5	0.0202	19 12 28	1	0.0040
24.1 33 10	1	0.0040	28 17 10	1	0.0040	14 20 16 18	2	0.0081	19 12 28.2	1	0.0040
24.1 36 10	1	0.0040	28 17 11	1	0.0040	14 20 17 16	1	0.0040	19 12 29	1	0.0040
25.1 18 10	1	0.0040	28 18 12	1	0.0040	14 20 17 16.2	2	0.0081	19 12 29.2	2	0.0081
25.1 19 11	2	0.0081	28 19 10	2	0.0081	14 20 17 17	4	0.0161	19 12 30.2	6	0.0242
25.1 19.1 11	1	0.0040	28 19 12	1	0.0040	14 20 17 18	1	0.0040	19 12 31	2	0.0081
25.1 20 10	1	0.0040	28 20 10	1	0.0040	14 20 18 16	2	0.0081	19 12 31.2	6	0.0242
25.1 21 10	3	0.0121	28 21 12	1	0.0040	14 20 18 16.2	1	0.0040	19 12 32	4	0.0161
25.1 22 10	2	0.0081	29 17 11	1	0.0040	14 20 18 17	2	0.0081	19 12 32.2	2	0.0081
25.1 22 12	2	0.0081	29 18 10	1	0.0040	14 20 18 17.2	1	0.0040	19 12 33	1	0.0040
25.1 22 9	1	0.0040	29 19 10	1	0.0040	14 20 19 16.2	1	0.0040	19 12 33.2	1	0.0040
25.1 22.1 10	1	0.0040				14 20 19 18	2	0.0081	19 13 28	1	0.0040
25.1 23 10	1	0.0040				14 21 15 18	1	0.0040	19 13 28.2	1	0.0040

25.1 23 11	1	0.0040	14 21 15.3 16	1	0.0040	19 13 29	1	0.0040
25.1 24 10	1	0.0040	14 21 16 17	1	0.0040	19 13 30	1	0.0040
25.1 24 9	1	0.0040	14 21 16 18	1	0.0040	19 13 30.2	1	0.0040
25.1 25 10	4	0.0161	14 21 17 15	1	0.0040	19 13 31	2	0.0081
25.1 26 10	3	0.0121	14 21 17 16.2	1	0.0040	19 13 31.2	8	0.0323
25.1 27 12	2	0.0081	14 21 17 17	4	0.0161	19 13 32	1	0.0040
25.1 28 11	1	0.0040	14 21 17 18	1	0.0040	19 13 32.2	2	0.0081
25.1 33 10	1	0.0040	14 21 18 17	2	0.0081	19 13 33	1	0.0040
25.1 36 11	1	0.0040	14 21 19 17.2	1	0.0040	19 13 34	1	0.0040
26.1 17 11	1	0.0040	14 21 19 18	1	0.0040	19 14 29.2	1	0.0040
26.1 18 10	2	0.0081	14 21 20 19	1	0.0040	19 14 30.2	3	0.0121
26.1 18 11	2	0.0081	14 22 16 17	1	0.0040	19 14 31.2	4	0.0161
26.1 19 10	2	0.0081	14 22 18 17	1	0.0040	19 14 32	1	0.0040
26.1 19 11	2	0.0081	14 23 16 17	1	0.0040	19 14 32.2	1	0.0040
26.1 20 10	4	0.0161	14 23 18 17.2	1	0.0040	19 15 33	1	0.0040
26.1 21 10	4	0.0161	15 15 19 17	1	0.0040	19 15 33.2	1	0.0040
26.1 21 11	2	0.0081	15 16 18 17	1	0.0040	20 11 32	1	0.0040
26.1 21 12	1	0.0040	15 17 15 17	1	0.0040	20 12 32	1	0.0040
26.1 21 13	1	0.0040	15 17 16 17	1	0.0040	20 12 33	1	0.0040
26.1 22 10	2	0.0081	15 17 17 16	1	0.0040	20 13 29.2	1	0.0040
26.1 22 11	1	0.0040	15 17 17 17	1	0.0040	20 13 31.2	1	0.0040
26.1 22 12	1	0.0040	15 17 17 18	1	0.0040	20 13 32.2	1	0.0040
26.1 23 10	3	0.0121	15 17 18 18	1	0.0040	20 13 33	1	0.0040
26.1 24 10	1	0.0040	15 18 16 16	1	0.0040	20 13 33.2	1	0.0040
26.1 25 10	1	0.0040	15 18 16 18	1	0.0040	20 14 30	2	0.0081
26.1 26 11	1	0.0040	15 18 17 17	1	0.0040	20 15 33	1	0.0040
26.1 28 11	1	0.0040	15 18 18 16.2	1	0.0040	21 11 30	1	0.0040
26.1 31 10	2	0.0081	15 18 18 17	1	0.0040	21 13 33.2	1	0.0040
27.1 18 10	1	0.0040	15 18 18 18	1	0.0040	21 15 30.2	2	0.0081
27.1 18 11	2	0.0081	15 18 19 18	1	0.0040			
27.1 19 11	2	0.0081	15 19 14 16	1	0.0040			
27.1 20 11	2	0.0081	15 19 15 16	1	0.0040			

27.1 21 10	2	0.0081	15 19 15 17	1	0.0040
27.1 21 12	1	0.0040	15 19 16 17	3	0.0121
27.1 22 10	3	0.0121	15 19 17 15	2	0.0081
27.1 23 11	1	0.0040	15 19 17 16	1	0.0040
27.1 24 11	1	0.0040	15 19 17 17	2	0.0081
27.1 25 10	1	0.0040	15 19 17 18	1	0.0040
27.1 25 11	2	0.0081	15 19 18 16.2	1	0.0040
27.1 26 12	1	0.0040	15 19 18 18	3	0.0121
27.1 27 10	1	0.0040	15 19 18 19	1	0.0040
27.1 27 11	1	0.0040	15 19 19 17	3	0.0121
27.1 27 12	1	0.0040	15 19 19 18	1	0.0040
27.1 28 11	1	0.0040	15 19 20 20	1	0.0040
27.1 29 10	1	0.0040	15 20 13 17	1	0.0040
27.1 29 11	2	0.0081	15 20 14 17	1	0.0040
27.1 31 11	1	0.0040	15 20 15 17	1	0.0040
27.1 34 10	1	0.0040	15 20 16 16	2	0.0081
28.1 16 11	1	0.0040	15 20 16 17	5	0.0202
28.1 18 11	1	0.0040	15 20 16.3 18	1	0.0040
28.1 19 9	1	0.0040	15 20 17 16	1	0.0040
28.1 20 10	1	0.0040	15 20 17 17.2	1	0.0040
28.1 21 10	1	0.0040	15 20 17 18	3	0.0121
28.1 21 12	2	0.0081	15 20 18 17	4	0.0161
28.1 22 11	1	0.0040	15 20 18 18	1	0.0040
28.1 23 10	3	0.0121	15 20 18 19	1	0.0040
28.1 23 13	1	0.0040	15 20 19 17	1	0.0040
28.1 28 11	1	0.0040	15 20 21 18	1	0.0040
28.1 28 12	1	0.0040	15 21 17 16	1	0.0040
28.1 29 10	1	0.0040	15 21 17 18	1	0.0040
28.1 29 11	1	0.0040	15 21 18 16.2	1	0.0040
28.1 30 10	1	0.0040	15 21 18 17	1	0.0040
28.1 30 11	1	0.0040	15 21 18 18	1	0.0040
28.1 32 10	2	0.0081	15 21 19 16	1	0.0040

28.2 19 10	1	0.0040	15 21 19 17	1	0.0040
29.1 18 10	1	0.0040	15 21 20 18	1	0.0040
29.1 19 10	1	0.0040	15 22 14 18	1	0.0040
29.1 19 12	1	0.0040	15 22 17 17	2	0.0081
29.1 21 10	1	0.0040	15 22 17 18	2	0.0081
29.1 22 11	1	0.0040	15 23 16 17	1	0.0040
29.1 23 10	1	0.0040	16 17 17 20	1	0.0040
29.1 23 11	1	0.0040	16 18 15 17	1	0.0040
29.1 24 10	1	0.0040	16 19 16 17	2	0.0081
29.1 24 11	2	0.0081	16 19 17 17	1	0.0040
29.1 26 10	1	0.0040	16 19 18 18	1	0.0040
29.1 27 10	1	0.0040	16 20 16 17	1	0.0040
29.1 32 11	2	0.0081	16 20 17 18	1	0.0040
30.1 18 10	1	0.0040	16 20 18 21.1	1	0.0040
30.1 20 10	2	0.0081	16 21 14 17	1	0.0040
30.1 20 11	1	0.0040	16 21 17 18	1	0.0040
30.1 20 9	1	0.0040	16 21 19 18	1	0.0040
30.1 21 11	1	0.0040	17 19 17 17	1	0.0040
30.1 21 12	1	0.0040	17 19 19 18	1	0.0040
31.1 21 10	1	0.0040	17 21 15 18	1	0.0040
31.1 23 10	1	0.0040	18 23 20 16	1	0.0040

LG1: DXS10148-DXS10135-DXS8378; LG2: DXS10159-DXS10162-DXS10164; LG3: DXS7132-DXS10079-DXS10074-DXS10075; LG4: DXS6809-DXS6789; LG5: DXS7424-DXS101; LG6: DXS10103-HPRTB-DXS10101; LG7: DXS10134-DXS7423.

Supplementary Table S10. The Nei's genetic distance between the Guizhou Gelao and other 13 neighboring populations based on genetic variations of 19 X-STRs

Populations	[01]	[02]	[03]	[04]	[05]	[06]	[07]	[08]	[09]	[10]	[11]	[12]	[13]	[14]
[01] Southern-Han														
[02] Tibet-Tibetan2	0.0304													
[03] Xinjiang-Uyghur2	0.0427	0.0410												
[04] Ningxia-Hui	0.0106	0.0304	0.0380											
[05] Tibet-Tibetan1	0.0243	0.0168	0.0399	0.0315										
[06] Xinjiang-Uygur1	0.0355	0.0446	0.0281	0.0381	0.0437									
[07] Guanzhong-Han	0.0099	0.0258	0.0376	0.0135	0.0217	0.0282								
[08] Xinjiang-Kazakh	0.0327	0.0301	0.0193	0.0296	0.0341	0.0181	0.0243							
[09] Xinjiang-Xibe	0.0206	0.0328	0.0416	0.0232	0.0271	0.0365	0.0142	0.0329						
[10] Liangshan-Yi	0.0175	0.0186	0.0407	0.0185	0.0202	0.0315	0.0099	0.0291	0.0211					
[11] Sichuan-Han	0.0095	0.0240	0.0404	0.0135	0.0198	0.0329	0.0089	0.0279	0.0189	0.0126				
[12] Sichuan-Tibetan	0.0283	0.0285	0.0519	0.0387	0.0239	0.0503	0.0253	0.0450	0.0330	0.0227	0.0257			
[13] Guizhou-Miao	0.0113	0.0324	0.0460	0.0171	0.0252	0.0325	0.0108	0.0357	0.0201	0.0150	0.0117	0.0317		
[14] Guizhou-Gelao	0.0093	0.0274	0.0394	0.0119	0.0235	0.0280	**0.0070**	0.0265	0.0200	0.0124	0.0093	0.0304	0.0075	

Supplementary Table S11. The Nei's genetic distances among 23 Chinese populations based on genetic variations of 11 overlapping X-STRs

Populations	[01]	[02]	[03]	[04]	[05]	[06]	[07]	[08]	[09]	[10]	[11]	[12]	[13]	[14]	[15]	[16]	[17]	[18]	[19]	[20]	[21]	[22]	[23]
[01] Guizhou-Gelao																							
[02] Guizhou-Miao	0.0087																						
[03] Southern-Han	0.0089	0.0106																					
[04] Tibet-Tibetan2	0.0268	0.0280	0.0228																				
[05] Xinjiang-Uyghur2	0.0390	0.0482	0.0448	0.0533																			
[06] Ningxia-Hui	0.0120	0.0179	0.0123	0.0293	0.0376																		
[07] Tibet-Tibetan1	0.0234	0.0281	0.0208	0.0146	0.0426	0.0299																	
[08] Xinjiang-Uyghur1	0.0305	0.0373	0.0374	0.0650	0.0327	0.0433	0.0564																
[09] Guanzhong-Han	0.0080	0.0127	0.0092	0.0284	0.0428	0.0131	0.0224	0.0331															
[10] Xinjiang-Kazakh	0.0247	0.0367	0.0349	0.0486	0.0194	0.0306	0.0405	0.0202	0.0262														
[11] Xinjiang-Xibe	0.0234	0.0257	0.0206	0.0390	0.0474	0.0244	0.0339	0.0439	0.0150	0.0373													
[12] Liangshan-Yi	0.0090	0.0141	0.0121	0.0149	0.0390	0.0129	0.0185	0.0387	0.0080	0.0276	0.0221												
[13] Sichuan-Han	0.0127	0.0129	0.0090	0.0231	0.0404	0.0129	0.0205	0.0366	0.0092	0.0277	0.0199	0.0118											
[14] Sichuan-Tibetan	0.0422	0.0429	0.0322	0.0303	0.0638	0.0438	0.0312	0.0711	0.0343	0.0585	0.0468	0.0283	0.0340										
[15] Northern-Han	0.0054	0.0070	0.0082	0.0243	0.0388	0.0129	0.0209	0.0321	0.0068	0.0259	0.0176	0.0082	0.0099	0.0357									
[16] Guangdong-Han	0.0089	0.0140	0.0085	0.0195	0.0473	0.0127	0.0241	0.0433	0.0115	0.0316	0.0292	0.0092	0.0130	0.0358	0.0093								
[17] Shanghai-Han	0.0059	0.0129	0.0118	0.0348	0.0451	0.0148	0.0282	0.0342	0.0107	0.0305	0.0268	0.0139	0.0151	0.0536	0.0087	0.0116							
[18] Henan-Han	0.0075	0.0078	0.0105	0.0255	0.0439	0.0169	0.0206	0.0344	0.0053	0.0282	0.0196	0.0090	0.0098	0.0368	0.0037	0.0131	0.0115						
[19] Liaoning-Korean	0.0066	0.0120	0.0112	0.0321	0.0472	0.0162	0.0272	0.0308	0.0051	0.0282	0.0165	0.0112	0.0111	0.0451	0.0074	0.0147	0.0086	0.0059					
[20] Guangdong-Zhuang	0.0118	0.0162	0.0111	0.0264	0.0430	0.0158	0.0253	0.0376	0.0117	0.0290	0.0224	0.0128	0.0113	0.0391	0.0109	0.0119	0.0143	0.0118	0.0134				
[21] Guangdong-Yao	0.0189	0.0229	0.0147	0.0348	0.0498	0.0213	0.0341	0.0437	0.0222	0.0405	0.0400	0.0226	0.0248	0.0421	0.0220	0.0184	0.0207	0.0240	0.0254	0.0192			
[22] Fujian-She	0.0141	0.0209	0.0220	0.0347	0.0609	0.0285	0.0329	0.0508	0.0215	0.0451	0.0390	0.0224	0.0287	0.0568	0.0162	0.0203	0.0183	0.0164	0.0170	0.0256	0.0293		
[23] Xinjiang-Uyghur3	0.0325	0.0358	0.0379	0.0472	0.0262	0.0350	0.0403	0.0315	0.0373	0.0233	0.0491	0.0388	0.0385	0.0685	0.0345	0.0360	0.0368	0.0380	0.0398	0.0409	0.0429	0.0503	
